# J-domain proteins cooperate with Hsp70 to drive multiphase separation of RNA-binding-deficient TDP-43

**DOI:** 10.1016/j.jbc.2025.110854

**Published:** 2025-10-22

**Authors:** Kian Hua Yeo, Jian Hua Kong, Qing Hao Ng, Mi-Jeong Yoon, Olivia Agatha, Eunyoung Chae, Hyun O Lee, H Shawn Je, Young-Jun Choe

**Affiliations:** 1 School of Biological Sciences, Nanyang Technological University, Singapore, Singapore; 2 Department of Biological Sciences, National University of Singapore, Singapore, Singapore; 3 Department of Biology, University of Oxford, Oxford, United Kingdom; 4 Department of Biochemistry, University of Toronto, Toronto, Ontario, Canada; 5 Neuroscience and Behavioral Disorders Programme, Duke-National University of Singapore Medical School, Singapore, Singapore

**Keywords:** molecular chaperone, TAR DNA-binding protein 43 (TDP-43), RNA-binding protein, heat shock protein 70 (Hsp70), heat shock protein 40 (Hsp40), J-domain protein (JDP), phase separation, amyotrophic lateral sclerosis (ALS), *Saccharomyces cerevisiae*, anisosome

## Abstract

RNA-free TDP-43, resulting from mutations or post-translational modifications in its RNA-binding domain, forms multiphase condensates with Hsp70 chaperones enriched in the core. The presence of this structure in the nucleus is thought to be associated with disease states. However, the mechanisms underlying its formation remain poorly understood. In particular, it is unclear whether J-domain proteins (JDPs), critical co-chaperones of Hsp70, are incorporated into multiphase condensates, and if so, how they contribute to the phase separation process. Using yeast as a model organism with a relatively small JDP family, we identified Sis1, but not Ydj1, as an important factor in TDP-43 multiphase separation. RNA-binding-deficient TDP-43 initially forms uniform condensates enriched with the JDP Sis1 but not with Hsp70. Subsequent recruitment of Hsp70 transforms these uniform structures into multiphase condensates, with both Sis1 and Hsp70 enriched in the core. This transition requires a functional J-domain, which stimulates Hsp70 ATPase activity. These findings reveal a key role for JDPs in TDP-43 multiphase separation and highlight JDP specificity in this process.

TAR DNA-binding protein 43 (TDP-43) is an RNA-binding protein that primarily functions in the nucleus to regulate RNA metabolism (1). However, its cytoplasmic aggregation is a hallmark of several neurodegenerative diseases, including amyotrophic lateral sclerosis (ALS), a subtype of frontotemporal lobar degeneration (FTLD), and limbic-predominant age-related TDP-43 encephalopathy (LATE) (2, 3, 4). TDP-43 aggregation is driven by amyloid fibrillation of its C-terminal low-complexity domain (LCD; amino acids 274–414 of the full 414-residue protein) (5, 6, 7, 8, 9, 10), which is enriched in glycine, serine, asparagine, and glutamine residues—together accounting for 64% of the domain’s composition. The LCD also exhibits phase separation behavior (11, 12, 13, 14), enabling TDP-43 to dynamically partition into RNA-enriched nuclear bodies under normal conditions (15, 16, 17). Under stress conditions, TDP-43 is also recruited to cytoplasmic stress granules (18, 19, 20, 21, 22). A recent study showed that the combination of oxidative stress and high local concentrations of TDP-43 within stress granules can trigger its aggregation (23).

Consistent with the phase-separating and amyloid-forming properties of the TDP-43 LCD, most ALS- and FTLD-associated mutations cluster within this domain (24, 25). However, several pathogenic mutations have also been identified within the RNA-binding domain, reducing the RNA-binding capacity of TDP-43 (26, 27, 28). Notably, this domain can also undergo post-translational modification by acetylation or SUMOylation, altering the function and behavior of TDP-43 (29, 30, 31, 32). Recent studies have shown that RNA-binding-deficient TDP-43 undergoes multiphase separation (33, 34, 35), forming a shell-like structure named the anisosome, which may contribute to the development of pathological aggregates (35). Notably, the inner core of these shell-like condensates is enriched in Hsp70 chaperones (35). However, whether Hsp70 co-chaperones are involved in this multiphase separation remains unknown.

Hsp70 proteins are a ubiquitous family of molecular chaperones that facilitate protein folding and prevent aggregation (36, 37, 38). They consist of an N-terminal nucleotide-binding domain (NBD) and a C-terminal substrate-binding domain (SBD). Substrate affinity is regulated by the nucleotide state of the NBD (39): when bound to ATP, the SBD adopts an open conformation with low substrate affinity (40). Hsp40 co-chaperones deliver nonnative polypeptides to ATP-bound Hsp70 and stimulate its ATPase activity through their J-domain (41, 42, 43). ATP hydrolysis in the NBD converts the SBD to a closed conformation, thereby trapping the transferred substrates (44). In this manner, Hsp70 prevents protein aggregation by binding to exposed hydrophobic motifs of nonnative substrates (45, 46). Nucleotide exchange factors then promote ADP-ATP exchange, reopening the SBD to release the substrates (47), which can subsequently proceed to folding.

Accumulating evidence indicates that Hsp70 can interact with various biomolecular condensates (48, 49, 50, 51). However, its selective enrichment within a subregion of condensates, thereby forming a distinctive core-shell architecture, is a rare and poorly understood phenomenon (35). In this study, we investigate the mechanism underlying the multiphase separation of TDP-43 (shell) and Hsp70 (core), with a particular focus on the role of Hsp40 co-chaperones (hereafter, referred to as J-domain proteins or JDPs). We demonstrate that JDPs cooperate with Hsp70 in this unique phase separation process, extending their role beyond classical protein folding. Using yeast as a model organism with a simplified JDP repertoire compared to that of humans (52), we show that Sis1 is the primary JDP for TDP-43 multiphase separation.

## Results

### RNA-binding-deficient TDP-43 forms shell-like condensates in yeast

**Figure 1:**
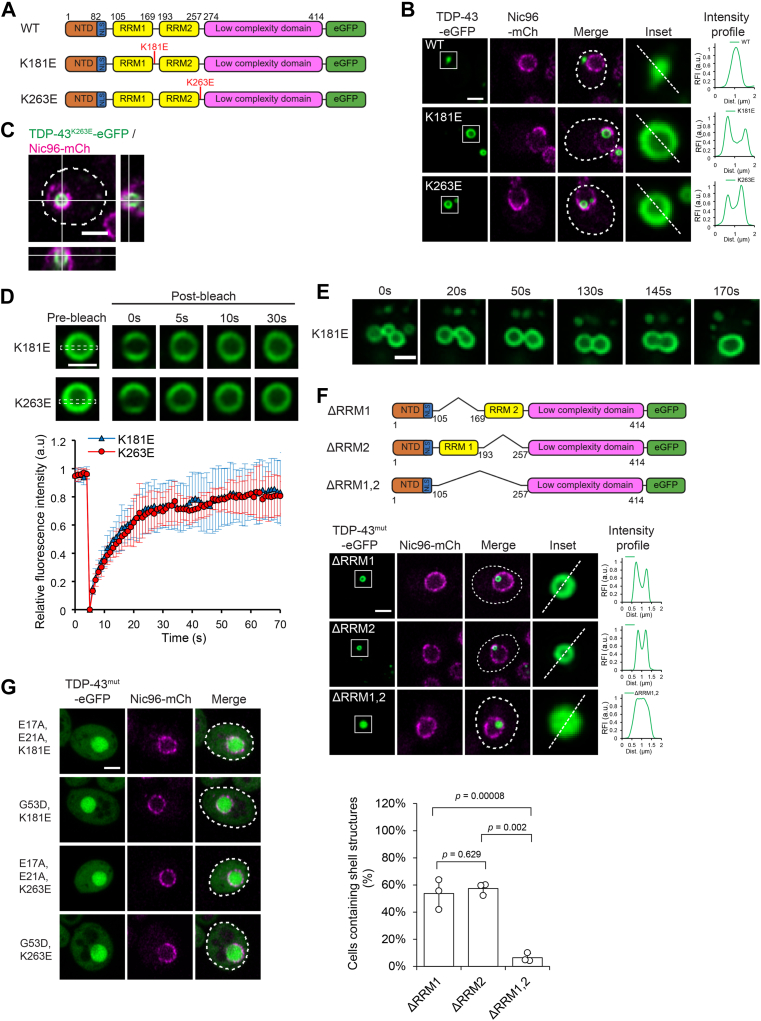
**RNA-binding-deficient TDP-43 forms shell-like condensates in yeast.***A*, schematic representation of WT TDP-43 and two RNA-binding-deficient TDP-43 mutants. *B*, WT and mutant TDP-43 proteins were expressed in WT yeast cells. mCherry-tagged Nic96, a component of the nuclear pore complex, was co-expressed as a nuclear marker. Dashed lines indicate cell outlines. Areas within *white squares* are enlarged in the insets and analyzed for intensity profiles. Fluorescence intensity profiles were obtained by measuring signal across the *white lines* shown in the insets and normalized to the maximum intensity. Scale bar, 2 μm. *C*, orthogonal projection of Z-stack images of a TDP-43^K263E^-eGFP condensate. The dashed line indicates a cell outline. Scale bar, 2 μm. *D*, FRAP analysis of TDP-43 shell-like condensates. Representative images of condensates before and after photobleaching are shown in the *top panel*. Dotted rectangles indicate the photobleached areas. Scale bar, 1 μm. Fluorescence recovery curves (mean ± SD; n = 10 condensates) are shown in the *bottom panel*. *E*, time-lapse imaging of a single cell containing multiple TDP-43^K181E^-eGFP shells. Scale bar, 1 μm. *F*, schematic of RRM-truncated TDP-43 mutants (*top*); representative micrographs of the truncated TDP-43 mutants expressed in WT yeast cells (*middle*); and quantification of shell formation (>300 cells counted, n = 3, *bottom*). Dashed lines in the merged micrographs indicate cell outlines. Areas within *white squares* are enlarged in the insets and analyzed for intensity profiles. Nic96-mCherry, nuclear marker. Scale bar, 2 μm. *G*, expression of RNA-binding-deficient TDP-43 mutants (K181E or K263E) carrying additional mutations in the NTD that abolish self-interaction. Dashed lines in the merged images indicate cell outlines. Nic96-mCherry, nuclear marker. Scale bar, 2 μm.

To determine whether yeast can serve as a model to study the mechanisms of TDP-43 multiphase separation, we first examined the behavior of the protein in this system. Human TDP-43 was tagged at its C-terminus with enhanced GFP (eGFP) (Fig. 1*A*). TDP-43 consists of an N-terminal domain (NTD) with a ubiquitin-like fold (53, 54), followed by a nuclear localization signal (NLS) (55, 56), two RNA recognition motifs (RRM1 and RRM2), and a C-terminal low-complexity domain (LCD). As previously reported (57), wild-type (WT) TDP-43 expressed under the control of the galactose-inducible *GAL1* promoter formed punctate structures in the cytoplasm, visualized outside the mCherry-labeled nuclear pore complex subunit Nic96 (Fig. 1*B*, top row). Consistent with the aggregating property of the LCD, its deletion abolished puncta formation, resulting in diffuse nuclear localization (Fig. S1*A*, ΔLCD) (57). The NTD alone was sufficient to direct the eGFP fusion protein to the nucleus without RNA recognition motifs (RRMs) (compare NTD and RRM in Fig. S1*A*) (57), indicating that the NLS of TDP-43 is functional in yeast cells.

We next examined whether the shell-like TDP-43 condensates, previously observed in human cells (Fig. S1*B*) (33, 34, 35), could also form in yeast, using the TDP-43^K181E^ and TDP-43^K263E^ mutants. These two mutations, located near RRM1 and RRM2 (Fig. 1*A*), have been identified in familial ALS and FTLD, respectively (26, 27), and impair the RNA-binding ability of TDP-43 (26). As expected, both TDP-43 mutants expressed in yeast formed intranuclear ring-like structures (Fig. 1*B*, second and third rows), which varied in size but were overall comparable to those observed in human cells (Fig. S1*C*). In addition to these rings, smaller and more uniform foci were also observed (Fig. S1*D*). The mutations did not affect TDP-43 expression levels in yeast (Fig. S1*E*). Orthogonal projections of Z-stack images demonstrated that the observed two-dimensional rings correspond to three-dimensional spherical shell-like structures (Fig. 1*C*). To assess whether the mutant TDP-43 assemblies exhibit liquid-like behavior, we measured protein mobility within the shell structures using fluorescence recovery after photobleaching (FRAP) (Fig. 1*D*). The rapid recovery of fluorescence in bleached regions indicated that the proteins remain highly mobile within the shells. Additionally, time-lapse imaging captured the fusion of individual shell structures into a single assembly while retaining a central hollow core (Fig. 1*E*), further supporting their dynamic and liquid-like properties. To test whether residual RNA interactions contribute to shell formation, we examined the TDP-43^5FL^ mutant, in which five phenylalanine residues critical for stacking with RNA bases were substituted with leucine, thereby abolishing RNA binding (Fig. S1*F*) (58, 59, 60, 61). Similar to TDP-43^K181E^ and TDP-43^K263E^, the RNA-binding-deficient TDP-43^5FL^ also formed intranuclear shells, indicating that residual RNA interactions are not required for shell formation.

To further investigate the role of the RRM domains in shell formation, we examined the effects of their deletion. Although the individual RRM1 and RRM2 domains can bind UG-rich RNA (59), their tandem arrangement enhances RNA-binding affinity (58, 60). Interestingly, variants lacking either RRM1 or RRM2 also formed intranuclear shell-like structures (Fig. 1*F*, ΔRRM1 and ΔRRM2), similar to those observed in the K181E and K263E point mutants. However, shell formation occurred at a very low frequency when both RRMs were deleted (Fig. 1*F*, ΔRRM1,2). Instead, the protein formed uniform, spherical assemblies with rapid diffusion dynamics, as measured by FRAP (Fig. S1*G*). TDP-43 dimerizes through self-interaction of its NTDs (62, 63). When mutations (E17A, E21A, or G53D)—previously shown to disrupt NTD-mediated interactions (35, 62)—were introduced, RNA-binding-deficient TDP-43 formed neither shell-like nor uniform assemblies; the proteins were diffusely localized in the nucleus (Fig. 1*G*). Overall, shell-like TDP-43 condensation in yeast closely resembled that observed in human cells. This process requires: (i) the LCD, (ii) NTD-mediated self-interaction, and (iii) loss of RRM function. Although RNA-binding activity needs to be suppressed for shell formation, the presence of the RRMs is important for organizing the condensates into shell-like architectures (Fig. 1*F*, ΔRRM1,2).

### The core of TDP-43 shells in yeast is enriched in Ssa-subfamily Hsp70s

**Figure 2:**
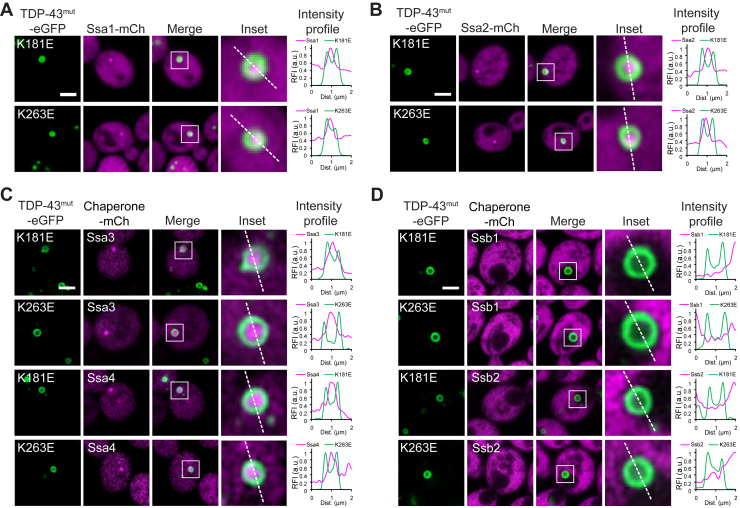
**TDP-43 shells in yeast are enriched with Ssa-subfamily Hsp70s in the core.** Co-expression of RNA-biding-deficient TDP-43 with mCherry-tagged Hsp70 chaperones: (*A*) Ssa1, (*B*) Ssa2, (*C*) Ssa3 and Ssa4, and (*D*) Ssb1 and Ssb2. Areas within *white squares* are enlarged in the insets and analyzed for intensity profiles. Scale bars, 2 μm.

In human cells, the core of TDP-43 shell structures is enriched with Hsp70 chaperones (Fig. S2*A*) (35). To investigate whether TDP-43 shells are organized similarly in yeast, we analyzed six Hsp70 family members known to function in both the cytosol and nucleus: Ssa1, Ssa2, Ssa3, Ssa4, Ssb1, and Ssb2. The chromosomal copies of each Hsp70 gene were C-terminally tagged with mCherry for microscopic analysis. Members of the Ssa subfamily—Ssa1 through Ssa4—were detected in both the cytosol and the nucleus (Fig. S2*B*). Notably, the stress-inducible isoforms Ssa3 and Ssa4 (64, 65) were expressed at markedly lower levels than the constitutively expressed Ssa1 and Ssa2 (Fig. S2*B*). We next expressed TDP-43^K181E^ and TDP-43^K263E^ in yeast strains carrying mCherry-tagged Hsp70 chaperones. Ssa1 and Ssa2 were strongly enriched in the core of TDP-43 condensates (Fig. 2, *A* amd *B*). In contrast, cytosolic WT TDP-43 foci (Fig. 1*B*) did not recruit Ssa1 or Ssa2 (Fig. S2*C*), and nuclear ΔRRM1,2 droplets (Fig. 1*F*) excluded these Hsp70 chaperones (Fig. S2*C*). Due to the low expression levels of Ssa3 and Ssa4 (Fig. S2*B*), their localization within TDP-43 shells was generally difficult to detect. However, in cells where these Hsp70s were stochastically expressed at higher levels, they were also enriched in the core of TDP-43 shell structures (Fig. 2*C*; see Fig. S2*D* for cell-to-cell variation in Ssa3 and Ssa4 levels). Ssb1 and Ssb2 were largely excluded from the nucleus at steady state (Fig. S2*E*), consistent with their known association with translating ribosomes in the cytosol (66, 67, 68). Although these chaperones may shuttle between the nucleus and cytosol to function in ribosome biogenesis (69, 70), their nuclear residence time is likely transient. Accordingly, Ssb1 and Ssb2 were not incorporated into nuclear TDP-43 shells (Fig. 2*D*). Taken together, TDP-43 shells formed in yeast can enrich all Ssa-subfamily Hsp70s within the core structure. Hereafter, these TDP-43 shell structures will be referred to as multiphase condensates, reflecting their distinct composition: a TDP-43-enriched shell and a chaperone-enriched core.

### JDP Sis1 is enriched in the core of TDP-43 multiphase condensates

**Figure 3:**
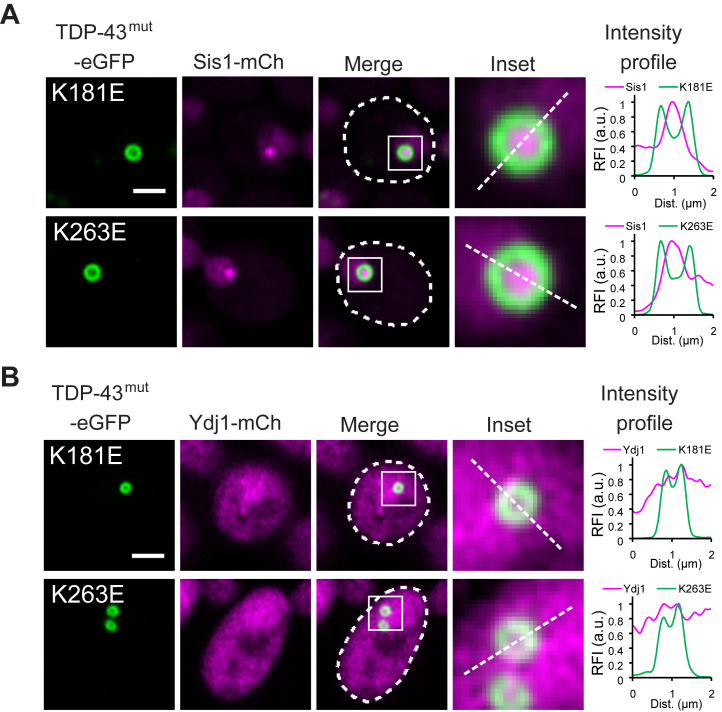
**JDP Sis1 is enriched in the core of TDP-43 multiphase condensates.** Co-expression of RNA-biding-deficient TDP-43 with mCherry-tagged JDPs: (*A*) Sis1 and (*B*) Ydj1. Dashed lines in the merged images indicate cell outlines. Areas within white squares are enlarged in the insets and analyzed for intensity profiles. Scale bars, 2 μm.

To investigate whether Hsp70 co-chaperones are also enriched in the core of TDP-43/Hsp70 multiphase condensates, we examined the localization of two major JDPs in yeast: Sis1 and Ydj1. When mCherry was fused to the C-terminus of each chromosomal gene, Sis1 was primarily localized to the nucleus, while Ydj1 was distributed across both the cytosol and nucleus (Fig. S3). Upon expression of RNA-binding-deficient TDP-43 mutants in these strains, Sis1 was strongly enriched in the core of TDP-43 condensates (Fig. 3*A*), whereas Ydj1 showed no such enrichment (Fig. 3*B*). These results indicate that not all JDPs interact equally with TDP-43 assemblies during multiphase separation. Ssa1 (Fig. 2*A*), Ssa2 (Fig. 2*B*), and Sis1 (Fig. 3*A*) were consistently enriched in the core of all TDP-43 shells, suggesting that these Hsp70 chaperones, and this specific JDP may constitute minimal components required for multiphase separation.

### Hsp70-stimulating activity of the Sis1 J-domain is required for TDP-43 shell–core separation

**Figure 4:**
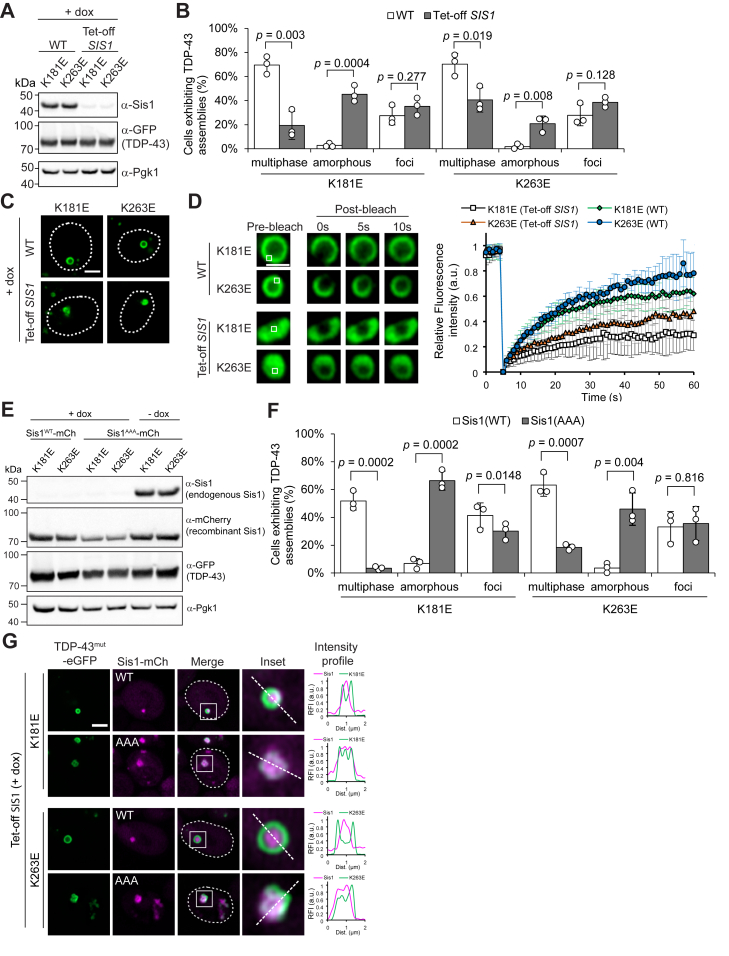
**Hsp70-stimulating activity of the Sis1 J-domain is required for TDP-43 shell–core separation.***A*, immunoblot analysis confirming Sis1 depletion upon doxycycline treatment. WT and Tet-off *SIS1* cells are genetically identical, except that the endogenous *SIS1* promoter in Tet-off *SIS1* cells was replaced with a doxycycline-repressible promoter. Cells were treated with doxycycline (10 μg/ml) for ∼20 h, followed by TDP-43 induction with galactose for 6 h. Pgk1 was used as a loading control (n = 3). *B*, quantitative analysis of RNA-binding-deficient TDP-43 assembly structures in cells depleted of Sis1. WT (*white* bars) and Tet-off *SIS1* cells (*gray* bars), treated with doxycycline as described in (*A*), were compared. Data are presented as mean ± SD; >300 cells counted across n = 3 independent experiments; *p*-values were obtained by two-tailed Student’s *t* test. *C*, representative images of shell and amorphous TDP-43 assemblies, taken from the experiment shown in (*B*). Dashed lines indicate cell outlines. Scale bar, 2 μm. *D*, FRAP analysis of multiphase and amorphous TDP-43 structures. Representative images of condensates before and after photobleaching are shown in the *left panel*. *White squares* indicate the photobleached regions. Scale bar, 1 μm. Fluorescence recovery curves (mean ± SD; n = 10 condensates) are shown in the right panel. *E*, Immunoblot analysis confirming depletion of endogenous Sis1 and expression of recombinant Sis1 (WT and AAA). Vectors expressing TDP-43 mutants and mCherry-tagged Sis1 (WT or AAA) were co-transformed into Tet-off *SIS1* cells. The last two lanes show endogenous Sis1 expression in the absence of doxycycline treatment. Pgk1 was used as a loading control (n = 3). *F*, quantitative analysis of microscopy data acquired under the same experimental conditions as in (*E*) (*left* four lanes). Data are presented as mean ± SD; n = 3 independent experiments; *p*-values were obtained by two-tailed Student’s *t* test. *G*, representative images of multiphase and amorphous TDP-43 assemblies, taken from the experiment shown in (*F*). Dashed lines in merged images indicate cell outlines. Areas within *white squares* are enlarged in the insets and analyzed for intensity profiles. Scale bar, 2 μm.

Having identified Sis1 in the core of TDP-43 shells, we next asked whether and how this JDP influences TDP-43 phase separation. To test this, we used a Tet-off yeast strain in which the endogenous *SIS1* promoter was replaced with a doxycycline-repressible promoter. Treatment with doxycycline effectively depleted Sis1 (Fig. 4*A*). Remarkably, Sis1 depletion led to a marked reduction in TDP-43 multiphase separation (Fig. 4*B*, *multiphase*). Instead, TDP-43^K181E^ and TDP-43^K263E^ assembled into irregularly shaped clumps (Fig. 4*B*, *amorphous*, and 4*C*), which were distinct from the smaller foci also observed under the same condition (Figs. 4*B*, *foci*, and S4*A*). Thus, small TDP-43 foci were consistently observed regardless of Sis1 presence (Fig. S1*D* and Fig. S4*A*). Notably, FRAP analysis showed that the mobility of TDP-43^K181E^ and TDP-43^K263E^ was significantly reduced in amorphous structures compared to multiphase condensates (Fig. 4*D*). Similar amorphous assemblies were also observed in WT cells, although they occurred at very low frequency (Fig. 4*B*, *amorphous*). Unlike the immobile amorphous structures in Sis1-depleted cells, these rare assemblies in WT cells remained dynamic (Fig. S4*B*). Although significantly reduced, multiphase condensates still formed under Sis1 depletion conditions (Fig. 4*B*), possibly through the activity of residual Sis1. These shell structures also remained dynamic (Fig. S4*C*). Overall, RNA-free TDP-43 assemblies in yeast appear to lose their dynamic properties when they fail to interact with Sis1. In contrast, depletion of another major JDP, Ydj1, had no effect on TDP-43 multiphase separation (Fig. S4, *D*–*F*), consistent with this co-chaperone not being enriched in TDP-43 shells (Fig. 3*B*). Together, these results demonstrate that Sis1 is the principal JDP for the formation of TDP-43/Hsp70 multiphase condensates in yeast.

To investigate the mechanistic role of Sis1 in TDP-43 multiphase separation, we disrupted its function to stimulate Hsp70 ATPase activity by mutating the conserved His-Pro-Asp (HPD) motif in the J-domain to Ala-Ala-Ala (AAA) (52), generating the Sis1^AAA^ mutant. We then compared the effects of recombinant Sis1^WT^ and Sis1^AAA^ in Tet-off *SIS1* cells. Upon doxycycline treatment, endogenous Sis1 was depleted, while mCherry-tagged recombinant Sis1^WT^ and Sis1^AAA^ were constitutively expressed from plasmids under the *SIS1* promoter (Fig. 4*E*). Notably, Sis1^WT^-mCherry restored the formation of TDP-43 multiphase condensates in Tet-off *SIS1* cells, whereas Sis1^AAA^-mCherry did not (Fig. 4*F*). Importantly, Sis1^AAA^-mCherry colocalized with the amorphous TDP-43 assemblies (Fig. 4*G*), indicating that physical association between Sis1 and TDP-43 is not sufficient to drive multiphase separation in the absence of Hsp70 ATPase stimulation. Sis1^AAA^ expression had a stronger inhibitory effect on TDP-43 shell formation than Sis1 depletion (compare Fig. 4, *B* and *F*). In Tet-off *SIS1* cells, residual Sis1 or partial compensation by other JDPs may account for the low level of shell formation. In contrast, Sis1^AAA^ likely competes with these functional JDPs for interaction with TDP-43, leading to a more pronounced disruption of the condensation process.

### TDP-43 assemblies transition from uniform to multiphase condensates in a concentration-dependent manner

**Figure 5:**
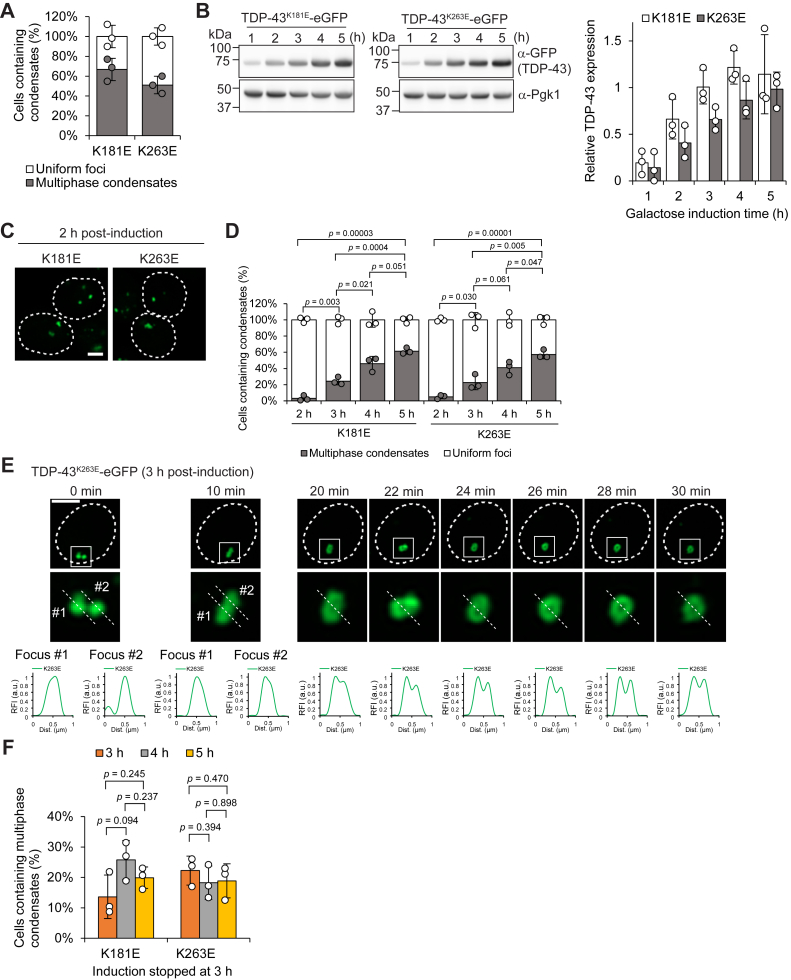
**TDP-43 assemblies transition from uniform to multiphase condensates in a concentration-dependent manner.***A*, quantification of cells exhibiting uniform foci or multiphase condensates after 6 h of galactose-induced TDP-43 mutant expression. Data are presented as mean ± SD; >300 cells counted across n = 3 independent experiments. *B*, immunoblot analysis confirming increasing levels of TDP-43 mutants over the course of galactose induction. Representative images of immunoblots are shown in the left panel. Pgk1 was used as a loading control. Quantitative analysis of band intensities is shown in the right panel. TDP-43-eGFP band intensities were normalized to Pgk1 band intensities. Data are presented as mean ± SD; n = 3 independent experiments. *C*, microscopy images of cells at early stages of condensation (2 h post-induction), displaying uniform foci. Dashed lines indicate cell outlines. Scale bar, 2 μm. *D*, Quantitative analysis of microscopy images collected over the course of galactose induction, showing the proportion of cells with multiphase condensates and uniform foci. Data are presented as mean ± SD; >300 cells counted across n = 3 independent experiments. *E*, time-lapse imaging of a single cell after 3 h of TDP-43^K263E^-eGFP induction. Dashed lines indicate cell outlines. Areas within white squares are enlarged in the insets and analyzed for intensity profiles. Scale bar, 2 μm. *F*, quantitative analysis of microscopy images showing the proportion of cells with multiphase condensates. Galactose induction was halted at 3 h by the addition of glucose, and cells were analyzed over the subsequent 2 h (5 h total). Data are presented as mean ± SD; n = 3 independent experiments; *p*-values were obtained by two-tailed Student’s *t* test.

As previously noted (Fig. S1*D*), both multiphase condensates and small foci were observed simultaneously when RNA-binding-deficient TDP-43 was expressed under the *GAL1* promoter for 6 h. Approximately 50 to 60% of cells displayed multiphase condensates (Fig. 5*A*, *Multiphase condensates*), which often coexisted with small uniform foci within the same cells (Fig. S5*A*). In the remaining cells, which did not exhibit multiphase condensates, only small uniform foci were observed (Fig. 5*A*, *Uniform foci*). Notably, no cells displayed diffuse localization of TDP-43^K181E^ or TDP-43^K263E^. The mixed population of small uniform foci and multiphase condensates prompted us to ask whether the small foci represent intermediate structures in the biogenesis of multiphase condensates. Taking advantage of the tightly regulated yet robust galactose-induction system in yeast, we monitored the assembly process of RNA-binding-deficient TDP-43 over time. Expression levels of the TDP-43 mutants gradually increased during galactose induction, reaching a plateau after approximately 4 to 5 h (Fig. 5*B*). At the early stage of expression (2 h post-induction), most cells displayed only small foci (Fig. 5*C*). As induction progressed, the proportion of cells with multiphase condensates gradually increased (Fig. 5*D*), while the number of cells containing only small foci declined, suggesting that TDP-43 foci may transition into multiphase condensates over time.

At the third hour of galactose induction, the number of cells displaying TDP-43 multiphase condensates had noticeably increased, although uniform foci remained predominant (Fig. 5*D*). We reasoned that the structural conversion of TDP-43 foci into multiphase condensates might be actively occurring at this stage. To capture these events, we randomly selected cells exhibiting uniform TDP-43 foci and successfully recorded their transition into shell structures (Figs. 5*E* and S5*B*). However, when TDP-43 expression was halted by adding glucose at the third hour of galactose induction, the frequency of TDP-43 shell formation did not increase over the subsequent 2 h (Fig. 5*F*). This contrasts sharply with the gradual rise in shell-containing cells observed under continuous galactose induction for 5 h (Fig. 5*D*, *multiphase*), indicating that the structural conversion of TDP-43 condensates depends on protein concentration rather than time alone. Consistently, higher expression of TDP-43 led to more frequent shell formation at earlier stages of galactose induction (Fig. S5, *C* and *D*). In summary, RNA-binding-deficient TDP-43 assembles into uniform foci at lower protein concentrations (Fig. 5, *B* amd *C*), but then transitions into multiphase condensates at higher levels of TDP-43 (Fig. 5*E*). While individual assemblies may grow by recruiting additional soluble TDP-43, the fusion of smaller foci—as observed in Figure 5*E* between 0 and 10 min—may provide an alternative mechanism for increasing droplet size and promoting structural conversion.

### Chaperones are sequentially recruited to enable the transition of TDP-43 condensates into multiphase structures

**Figure 6:**
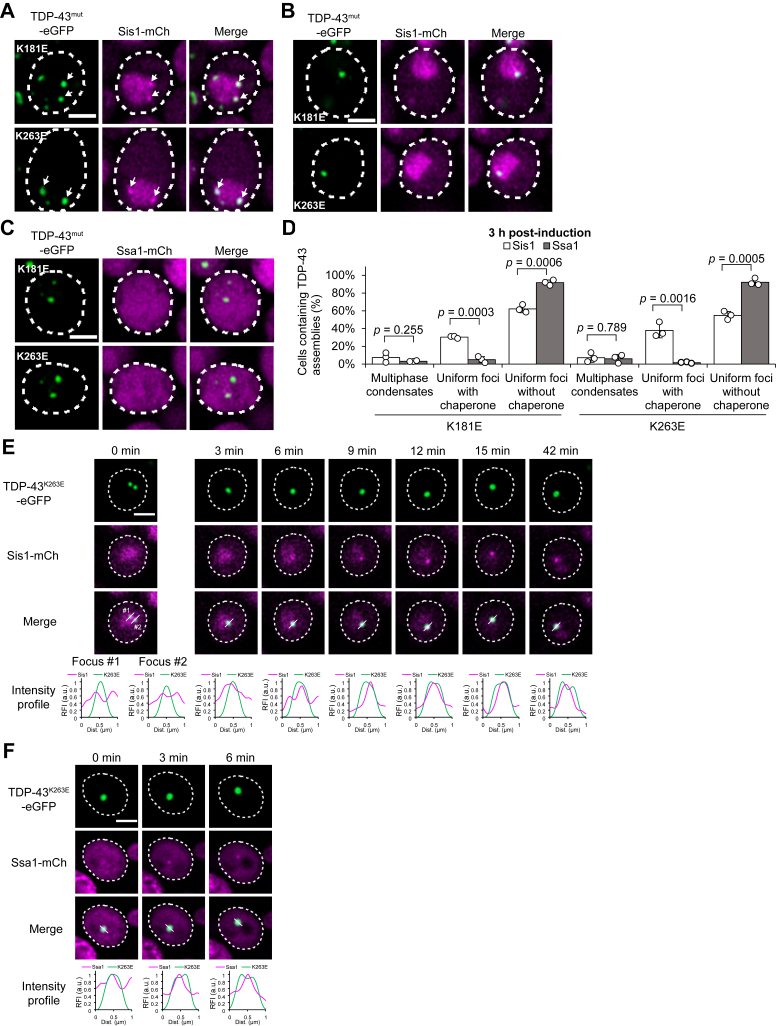
**Chaperones are sequentially recruited to enable the transition of TDP-43 condensates into multiphase structures.***A*, microscopy images of TDP-43^mut^-eGFP uniform foci were analyzed to examine colocalization with Sis1. TDP-43^mut^-eGFP was expressed for 3 h in galactose media in cells with genomic *SIS1* tagged with mCherry at the C-terminus. Arrows indicate foci that colocalize with Sis1-mCherry. Dashed lines indicate cell outlines. Scale bar, 2 μm. *B*, microscopy images of cells displaying TDP-43^mut^-eGFP foci lacking colocalization with Sis1. Dashed lines indicate cell outlines. Scale bar, 2 μm. *C*, microscopy images of cells displaying TDP-43^mut^-eGFP foci lacking colocalization with Ssa1. Dashed lines indicate cell outlines. Scale bar, 2 μm. *D*, quantitative analysis of microscopy data acquired under the same experimental conditions as shown in panels (*A-C*). Data are presented as mean ± SD; n = 3 independent experiments; *p*-values were obtained by two-tailed Student’s *t* test. *E*–*F*, Time-lapse imaging of TDP-43^K263E^-eGFP foci expressed in cells with genomic *SIS1* (*E*) or *SSA1* (*F*) tagged with C-terminal mCherry. TDP-43^K263E^-eGFP was expressed for 3 h in galactose media. Dashed lines indicate cell outlines. Intensity profiles along the white lines in the merged images are shown. Scale bar, 2 μm.

Having observed the morphological transition of TDP-43 condensates, we next examined the timing of Hsp70 Ssa1 and JDP Sis1 localization during this process. To this end, RNA-binding-deficient TDP-43 mutants were expressed in yeast strains expressing either Ssa1-mCherry or Sis1-mCherry. Chaperone localization was examined after 3 h of galactose induction, when uniform TDP-43 foci were the predominant assembly type (Fig. 5*D*). Despite their similar appearance, these foci fell into two distinct categories: those that colocalized with Sis1 (Fig. 6*A*) and those that did not (Fig. 6*B*). In stark contrast, Ssa1 was not enriched in the vast majority of TDP-43 foci (Fig. 6*C*). Although expression of TDP-43 mutants induced Ssa1 puncta in a subset of cells (Fig. S6*A*), these puncta did not colocalize with TDP-43 foci (Fig. S6*B*). The formation of Ssa1 puncta likely reflects proteotoxic stress caused by TDP-43^K181E^ and TDP-43^K263E^.

We next compared early-phase TDP-43 foci at 3 h post-induction with late-phase TDP-43 foci at 6 h (Fig. 6*D* *versus* Fig. S6*C*; see also Fig. S1*D* for representative late-phase foci). Notably, the colocalization patterns of Sis1 (white bars) and Ssa1 (gray bars) remained largely unchanged over time. Sis1 continued to partition into a subset of TDP-43 foci, whereas Ssa1 colocalization remained minimal even after prolonged induction. Thus, chaperone colocalization analysis reveals that uniform and multiphase condensates differ not only in structure but also in composition. While uniform TDP-43 foci do not enrich Ssa1, multiphase condensates consistently contain both Ssa1 and Sis1.

**Figure 7:**
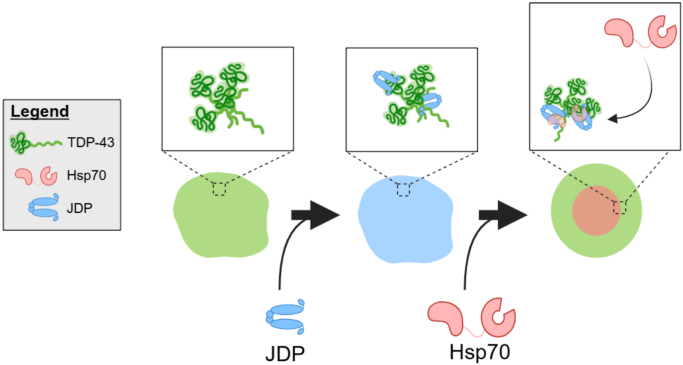
**Schematic model illustrating the multiphase separation of TDP-43 and Hsp70/JDP chaperones.** RNA-free TDP-43 forms uniform condensates that sequentially recruit JDP and Hsp70. A high concentration of TDP-43, enrichment of JDP and Hsp70, and cooperation between the two chaperones *via* the J-domain are required for the transition from uniform foci to shell-like multiphase condensates.

We next asked whether the heterogeneity among TDP-43 foci—with or without the JDP Sis1 (Fig. 6, *A versus B*)—represented distinct assembly types or reflected delayed Sis1 recruitment. To address this, we tracked randomly selected TDP-43 foci that initially showed no detectable colocalization with Sis1-mCherry (Fig. 6*E*). Z-stack analysis at 0 min confirmed the uniform morphology of the assemblies and the absence of Sis1-mCherry enrichment (Fig. S6*D*). Over time, we observed the accumulation of Sis1-mCherry within a TDP-43 focus (Figs. 6*E* and S6*D*, 6 and 9 min), followed by its transformation into a multiphase condensate in which Sis1 localized to the core (Figs. 6*E* and S6*D*, 42 min). We also tracked uniform TDP-43 foci that initially showed no detectable enrichment of Hsp70 Ssa1-mCherry (Fig. 6*F*). In contrast to Figure 6*E*, where Sis1 accumulation preceded structural transformation of a TDP-43 condensate, Ssa1 enrichment occurred almost concomitantly with changes in condensate structure, as indicated by the split of green peaks in the fluorescence intensity profile (Fig. 6*F* and S6*E*, 3 min). This difference between Sis1 and Ssa1 may explain their distinct steady-state colocalization with uniform TDP-43 foci (Fig. 6*D*). Taken together, our colocalization data indicate that the JDP Sis1 partitions into uniform TDP-43 condensates at an early stage. Subsequent enrichment of Hsp70 Ssa1 at later stages may trigger the structural conversion of RNA-binding-deficient TDP-43 condensates into multiphase assemblies, consisting of a TDP-43 shell and a chaperone-rich core (Fig. 7). This process requires a functional J-domain, which can stimulate Hsp70 ATPase (Fig. 4*G*).

### TDP-43 multiphase separation is severely impaired in *sse1*Δ yeast cells

Having identified the importance of JDPs in TDP-43 multiphase separation, we next asked whether nucleotide exchange factors (NEFs), the other essential cofactors in the Hsp70 ATPase cycle, also contribute to this process. To address this, we examined TDP-43 shell formation in yeast strains lacking individual nucleocytoplasmic NEFs: Sse1, Sse2, Fes1, and Snl1 (71). Notably, the RNA-binding-deficient TDP-43 mutants failed to form shells in *sse1*Δ cells (Fig. S7*A*). However, their expression levels were noticeably lower in *sse1*Δ compared with WT cells (Fig. S7*B*), which could potentially account for the absence of shells. To test this, we introduced two copies of the expression vectors, restoring TDP-43 levels nearly to those observed in WT cells (Fig. S7*C*). Despite this increase, TDP-43 shells were virtually undetectable in *sse1*Δ cells (Fig. S7*D*), indicating that the defect in multiphase separation cannot be explained by reduced protein expression. We next examined whether Sse1 itself was enriched in the core of TDP-43 shells, using a strain in which the genomic *SSE1* locus was fused to C-terminal mCherry. Unlike Hsp70s such as Ssa1 and Ssa2, or the JDP Sis1, Sse1 did not accumulate in the core of TDP-43 shells (Fig. S7*E*). Overall, our findings indicate that the full Hsp70 ATPase cycle, coordinated by both JDPs and NEFs, is required for TDP-43 multiphase separation. However, NEFs are unlikely to serve as structural elements of the condensate core, in contrast to Hsp70s and JDPs.

## Discussion

Recent studies have reported diverse forms of multiphase separation both *in vitro* (72, 73, 74, 75) and in cells (76, 77, 78, 79, 80, 81). In some cases, condensates can entrap the surrounding dilute phase, enabling even a single biopolymer to form multiphase structures with hollow cores (73). However, most cellular multiphase condensates likely contain internal dense phases composed of distinct biomolecules. The nucleolus is perhaps the most extensively studied example of such condensates (82), comprising three distinct compartments: the fibrillar center (FC), dense fibrillar component (DFC), and granular component (GC). These subcompartments support sequential stages of ribosome biogenesis, including rRNA transcription, rRNA processing and modification, and ribosome assembly. Key protein components of the DFC and GC—fibrillarin and NPM1, respectively—can undergo homotypic phase separation *in vitro*, with their condensation further enhanced by rRNA, another major nucleolar constituent (78, 83, 84). Purified fibrillarin and NPM1 can also undergo multiphase separation (78), recapitulating the layered organization of the nucleolus. Compared to the nucleolus and other known multiphase condensates, the incorporation of Hsp70 as an internal dense phase within the TDP-43 shell is particularly intriguing. Unlike phase-separating proteins such as fibrillarin and NPM1, which localize to specific cellular compartments and interact with defined molecular partners, Hsp70 is a ubiquitous chaperone capable of engaging a broad range of client proteins throughout the cell. This suggests that Hsp70-centered shell structures may not be unique to TDP-43, but could potentially form with other phase-separating proteins. Indeed, a recent study identified a similar condensate composed of a REV-ERBα shell and an Hsp70-enriched core (85). However, no previous studies have reported the presence of Hsp70 co-chaperones within the condensate core, raising the question of whether JDPs contribute to this unique multiphase organization. Notably, using yeast, which possesses a simpler JDP repertoire than human cells, this study demonstrates that the JDP Sis1 is not only enriched in the core of TDP-43 condensates but also required for robust TDP-43/Hsp70 multiphase separation.

Based on our observations, we propose a model describing how TDP-43, Hsp70, and JDPs assemble into a unique core-shell structure (Fig. 7). In the absence of RNA, TDP-43 exhibits enhanced phase separation, suggesting that RNA may function as a chaperone to suppress condensation (86, 87). This phase separation depends on self-association mediated by the N-terminal domain of TDP-43. The resulting uniform condensates initially recruit JDPs, but not Hsp70. Over time, the size and mass of these assemblies likely increase through continued recruitment of TDP-43 or by fusion between droplets. Once partitioned into these condensates, JDPs may help maintain their liquid-like properties during this stage. Supporting this, depletion of the JDP Sis1 reduces the fluidity of TDP-43 assemblies. Elevated local concentrations of JDPs may subsequently facilitate the partitioning of Hsp70 into uniform TDP-43 assemblies. This sequential recruitment of JDPs and Hsp70 is reminiscent of their cooperative mechanism during protein folding. Notably, both JDPs and Hsp70 have been shown to form liquid-like droplets *in vitro* in the presence of molecular crowding agents (48, 88). However, it remains unclear whether these chaperones can undergo phase separation in the cellular environment, where unfolded protein substrates are continuously generated by translating ribosomes. In this context, phase separation of RNA-binding-deficient TDP-43 may create a secluded environment that facilitates the local concentration of JDPs, followed by Hsp70. Once these chaperones accumulate beyond their critical concentration, they may undergo phase separation within TDP-43 droplets (89). For this secondary phase separation to occur, the interaction affinity between chaperones would need to exceed that between the chaperones and TDP-43. How such an affinity shift is achieved, and whether regulation of the Hsp70 ATPase cycle by JDPs and NEFs plays a role in this process, remain important questions for future investigation.

Overall, this study establishes yeast as a valuable model system for investigating chaperone-mediated multiphase separation. Partitioning of Hsp70 and/or JDPs into biomolecular condensates has been previously reported (48, 88, 90, 91, 92, 93), where these chaperones are thought to promote condensate dissolution or prevent their transition to solid-like states (94, 95). In contrast, our findings reveal a distinct effect of partitioned Hsp70 and JDPs: driving a structural transition from uniform to multiphase condensates. Although we anticipate that RNA-free TDP-43 undergoes a similar multiphase separation process in both yeast and human cells, the expanded JDP family in humans may lead to functional redundancy, potentially obscuring the contributions of individual JDPs to TDP-43 multiphase separation—a possibility that warrants further investigation.

## Experimental procedures

### Human cell lines

HEK293T cells were obtained from ATCC. The cell line was cultured in DMEM (Gibco, 11960044) supplemented with 10% FBS (GE HyClone, SV30160.03), 1X GlutaMax (Gibco, 3505061), 100 U/ml penicillin and 100 μg/ml streptomycin (Gibco, 15140122) at 37 °C in a humidified atmosphere containing 5% CO_2_. Plasmid transfection was performed using Lipofectamine 3000 (Invitrogen, L3000015) into 70% confluent cell culture.

### Yeast strains, media, and culture conditions

*Saccharomyces cerevisiae* strain BY4741 (*MATa his3Δ1 leu2Δ0 met15Δ0 ura3Δ0*) was used in all experiments. Cells were cultured at 30 °C in either 2X YPD medium (2% Bacto-yeast extract, 4% Bacto-peptone, and 4% glucose) or in synthetic dropout (SD) media. SD media consisted of 0.67% nitrogen base without amino acids (Sigma, Y0626), 2% carbon source (glucose, raffinose, or galactose) and was supplemented with the following amino acids: 0.04 g/L adenine sulfate, 0.04 g/L L-tryptophan, 0.02 g/L L-arginine hydrochloride, 0.03 g/L L-tyrosine, 0.03 g/L L-lysine hydrochloride, 0.05 g/L L-phenylalanine, 0.1 g/L L-glutamic acid, 0.1 g/L L-asparagine, 0.15 g/L L-valine, 0.2 g/L L-threonine, 0.375 g/L L-serine, 0.02 g/L L-methionine, 0.02 g/L uracil, 0.02 g/L L-histidine hydrochloride, 0.06 g/L leucine. Where indicated, doxycycline (Sigma, D9891) was added to a final concentration of 10 μg/ml. Yeast deletion mutants in the BY4741 background (*sse1*Δ, *sse2*Δ, *fes1*Δ, and *snl1*Δ) were obtained from the Yeast Knockout (YKO) Collection (Horizon Discovery).

### Yeast transformation

Yeast plasmid transformations were performed using the lithium acetate/single-stranded carrier DNA/polyethylene glycol (LiAc/SS carrier DNA/PEG) method (96). Briefly, cells were grown to logarithmic phase, harvested by centrifugation, and washed twice with Milli-Q water. The cell pellet was resuspended in a transformation mix containing LiAc (Sigma, 62393), ssDNA (Sigma, D1626), PEG 3350 (Sigma, P4338), and the plasmid of interest. Cells were heat-shocked at 42 °C for 20 min and plated onto the appropriate selection medium.

### Construction of yeast expression plasmids

To construct yeast expression plasmids, the human TDP-43 coding sequence was PCR-amplified from cDNA using the following primers: 5′-CCCGTCTAGAATGTCTGAATATATTCGGG-3′ (forward primer for WT TDP-43) and 5′-CCCGGGATCCCATTCCCCAGCCAGAAGAC-3′ (reverse primer for WT TDP-43). The PCR product was digested with XbaI and BamHI, and ligated into the pRS415GAL1-eGFP or pRS416GAL1-eGFP vector, which contains the *GAL1* promoter and an enhanced GFP (eGFP) sequence for C-terminal fusion to generate pRS415/6GAL1-TDP-43-eGFP. To generate the expression plasmid for *SIS1*, the genomic sequence of *SIS1*, including its native promoter, was PCR-amplified from yeast genomic DNA using the following primers: 5′-CCCGGAGCTCCAATCAGAACTCCTTTTTTATATG-3′ (forward primer for WT *SIS1*) and 5′-CCCGGGATCCAAAATTTTCATCTATAGCACG-3′ (reverse primer for WT *SIS1*). The PCR product was digested with SacI and BamHI and ligated into the pRS413-mCherry vector to generate pRS413-SIS1-mCherry. Similarly, for the *NIC96* expression plasmid, the genomic *NIC96* sequence, including its native promoter, was amplified from yeast genomic DNA using the following primers: 5′-CCCGGAGCTCTCTGTCGCAAGGACTATACG-3′ (forward primer for *NIC96*) and 5′-CCCGGGATCCTAGAGAGACGTCTATATTAATTAAAG-3′ (reverse primer for *NIC96*). The PCR product was digested with SacI and BamHI and ligated into either pRS413-mCherry or pRS416-GFP to generate pRS413NIC96-mCherry or pRS416NIC96-GFP, respectively.

### Site-directed mutagenesis

RNA-binding-deficient TDP-43 mutants (K181E and K263E) were generated by overlapping PCR. For each mutation, two DNA fragments were PCR-amplified from WT TDP-43 cDNA. The first fragment was amplified using the forward primer for WT TDP-43 and a mutation-specific reverse primer: 5′-CTTGGCTTTGCTCAGAATTAGGAAG-3’ (K181E reverse primer) or 5′-ATGCCGAACCTGAGCACAATAGC-3’ (K263E reverse primer). The second fragment was amplified using the reverse primer for WT TDP-43 and a mutation-specific forward primer complementary to the mutated region: 5′-CTTCCTAATTCTGAGCAAAGCCAAG-3′ (K181E forward primer) or 5′-GCTATTGTGCTCAGGTTCGGCATTG-3′ (K263E reverse primer). The two overlapping fragments were then used as templates for a final PCR to generate full-length TDP-43 carrying either the K181E or K263E mutation. The resulting PCR amplicons were digested with XbaI and BamHI and ligated into the pRS415GAL1-eGFP vector to generate pRS415GAL1-TDP43(K181E/K263E)-eGFP. Using the same restriction enzyme sites, the TDP-43^5FL^ mutant, containing five point mutations (F147L, TTT→TTA; F149L, TTC→TTA; F194L, TTC→TTG; F229L, TTT→CTT; and F231L, TTT→CTT), was ligated into the pRS415GAL1-eGFP vector.

To generate the *sis1*^*AAA*^ mutant, two overlapping DNA fragments were PCR-amplified using WT *SIS1* DNA as the template. The first fragment was amplified using the forward primer for WT *SIS1* and a mutation-containing reverse primer: 5′-CTTTTCTGTGTCACCTGTTGGCTTAGCTGCAGCATATTTTAGAGCTGCTTTTC-3′ (*sis1*^*AAA*^ reverse primer). The second fragment was amplified using the reverse primer for WT SIS1 and a mutation-containing forward primer complementary to the mutated region: 5′-GAAAAGCAGCTCTAAAATATGCTGCAGCTAAGCCAACAGGTGACACAGAAAAG-3′ (*sis1*^*AAA*^ forward primer). The overlapping fragments were used as templates for PCR amplification of full-length *sis1*^*AAA*^. The resulting PCR amplicons were digested with SacI and BamHI and ligated into the pRS413-mCherry vector to generate pRS413-*sis1*^*AAA*^-mcherry.

### Construction of yeast strains

All yeast strains used in this study were derived from the BY4741 background. The generation of yeast strains expressing mCherry-tagged chaperone (Ssa1-mCh, Ssa2-mCh, Sis1-mCh, and Ydj1-mCh) has been described previously (97). Additional strains used in this study (Ssa3-mCh, Ssa4-mCh, Ssb1-mCh, Ssb2-mCh, and Sse1-mCh) were generated using a similar approach. Briefly, the mCherry coding sequence was PCR-amplified using the following primers: 5′-CCCGAAGCTTGTGAGCAAGGGCGAG-3′ (forward primer excluding start codon) and 5′-CCCGAGATCTATATTACCCTGTTATCCCTAG-3′ (reverse primer including the ADH1 terminator). The PCR product was digested with HindIII and BglII and ligated into the corresponding sites of the pFA6a-His3MX6 vector to generate pFA6a-mCherry-His3.

This construct served as the template for generating gene-specific integration cassettes *via* PCR. Each cassette was designed to insert mCherry at the C-terminus of the endogenous locus *via* homologous recombination. The following primer pairs were used for amplification of the integration cassettes: 5′-ACGGGAGGTGGAGAAGATACAGGTCCAACAGTGGAAGAGGTTGATGTGAGCAAGGGCGAGGAGATAAC-3′ (SSA3 forward primer) and 5′-GGTTAAACATAAAAAGTAGCTAAATAGAACACTATAGAAGAATAACAGTATAGCGACCAGCATTC-3′ (SSA3 reverse primer); 5′-CCCACTGGAGCACCAGACAACGGCCCAACGGTTGAAGAGGTTGATGTGAGCAAGGGCGAGGAGGATAAC-3′ (SSA4 forward primer) and 5′-ACTAAGAAATTCGATGCTGCTACTTCATCGCATCTTTGTATTTATCAGTATAGCGACCAGCATTC-3′ (SSA4 reverse primer); 5′-GAAGTTGGTTTGAAGAGAGTTGTCACCAAGGCCATGTCTTCTCGTGTGAGCAAGGGCGAGGAGGATAAC-3′ (SSB1 forward primer) and 5′-ATATACAATATAAGTAATATTCATATATATGTGATGAATGCAGTCCAGTATAGCGACCAGCATTC-3′ (SSB1 reverse primer); 5′-GAAGTTGGTTTGAAGAGAGTTGTCACCAAGGCCATGTCTTCTCGTGTGAGCAAGGGCGAGGAGG-3′ (SSB2 forward primer) and 5′-AATGAAAAATATATATATGTGTATAACCTTAACCAGAATGACATCCAGTATAGCGACCAGCATTC-3′ (SSB2 reverse primer). 5′-AAGAAGGAAGAAAAGAAGGACACTGAAGGTGATGTTGACATGGACGTGAGCAAGGGCGAGGAGGATAAC-3′ (SSE1 forward primer) and 5′-AAAAAACAATAAAGATCCTTTTCTAGTTACTTTGCTGCATTAACACAGTATAGCGACCAGCATTC-3′ (SSE1 reverse primer). PCR amplicons were introduced into yeast cells by the standard transformation protocol, and successful integration clones were selected on SD media lacking histidine.

Tet-off *SIS1* and its parental strain R1158 were obtained from the yeast Tet-promoters Hughes Collection (yTHC, Horizon Discovery). To introduce the Tet-off promoter upstream of the *YDJ1* coding sequence, the KanMX-tetO_7_-TATA_*cyc1*_ cassette (98) was transformed into the R1158 strain. Transformants were selected on YPD plates supplemented with G418. Replacement of the endogenous promoter with the Tet-off promoter was confirmed by immunoblotting, which showed reduced Ydj1 protein levels following doxycycline treatment.

### Immunoblotting

Cells induced to express TDP-43 were incubated at 30 °C. An amount of culture equivalent to OD_600_ = 3 was harvested by centrifugation at 10,000*g* for 3 min. Cell pellets were resuspended in 12% trichloroacetic acid (TCA) solution, flash-frozen in liquid nitrogen and stored overnight at −80 °C. To prepare protein samples, cells were pelleted again, and the supernatant was removed. Pellets were washed with 400 μl cold acetone and re-pelleted by centrifugation. The acetone-washed pellets were air-dried on the benchtop for at least 6 h.

Protein pellets were resuspended in Laemmli buffer supplemented with β-mercaptoethanol and heated at 95 °C for 5 min. Samples were separated by SDS-PAGE using a Bis-Tris gel (4% stacking gel and 12% resolving gel) in MES running buffer (50 mM MES, 50 mM Tris, 1 mM EDTA, 0.1% SDS and 5 mM sodium bisulfite), then transferred onto 0.2 μm PVDF membrane using transfer buffer (25 mM Tris, 192 mM glycine, 10% methanol). Membranes were blocked in 3% milk (Millipore, 70166) for 1 h at room temperature, incubated with primary antibodies overnight at 4 °C, and then with secondary antibodies for 1 h at room temperature. Detection was performed using the SuperSignal West Pico PLUS Chemiluminescent Substrate (Thermo Fisher Scientific, CAT #34580), and membranes were imaged using a ChemiDoc MP imaging system (Bio-Rad).

The primary antibodies used were anti-GFP (Merck, CAT #11814460001; WB dilution 1:1000), anti-mCherry (Invitrogen, CAT #PA5-34974; WB dilution 1:1000), anti-SIS (Cosmo Bio USA, COP-080051; WB dilution 1:5000), anti-YDJ1 (Sigma -Aldrich, CAT #SAB5200007; WB dilution 1:2000) and anti-PGK1 (Invitrogen, CAT #459250; WB dilution 1:3000); anti-RPS3 (HAEL Ltd, HaB 1802; WB dilution 1:3000). The secondary antibodies used were HRP-conjugated anti-rabbit (Cell Signaling Technology, 7074S; WB dilution 1:3000) and HRP-conjugated anti-mouse (Cell Signaling Technology, 7076S; WB dilution 1:3000).

### Fluorescence microscopy

Confocal images were acquired using a ZEISS LSM 980 microscope equipped with Airyscan two and a Plan-Apochromat 63× oil immersion objective with a numerical aperture of 1.4. eGFP/GFP and mCherry/mRuby2 fluorescence were excited using 488 nm and 561 nm laser lines, respectively. Images were captured and processed using Zen Blue software (Carl Zeiss) and further analyzed using ImageJ software. Yeast cells were cultured in SD medium at 30 °C to logarithmic phase (OD_600_ < 1) and harvested after the specified duration of galactose induction, as detailed in each experiment. Before imaging, cells were suspended in SD medium, placed onto glass coverslips (Marienfeld Superior, CAT #0112700), and immobilized with a 1% agarose pad.

### Analysis of TDP-43 assembly morphology

Live-cell imaging was performed as described under Fluorescence microscopy. To distinguish between TDP-43 uniform foci and shell-like condensates, fluorescence intensity measurements were performed along a defined line indicated in the corresponding figures. Fluorescence intensities were normalized to the maximum value within each measurement and plotted as relative fluorescence intensity (RFI) profiles. Z-stack images were acquired at 0.2 μm intervals and used to generate an orthogonal projection of the TDP-43 shell-like structure.

To assess the effect of Sis1^WT^/Sis1^AAA^ on TDP-43 multiphase condensation, TDP-43-positive cells were categorized into three groups based on the morphology of TDP-43 assemblies: (1) cells containing shell-like structures, (2) cells containing amorphous structures, and (3) cells containing uniform foci. In this study, we defined shell-like structures by the presence of a visible hollowed core; amorphous assemblies as irregularly shaped structures larger than a 0.7 μm × 0.7 μm square; and structures smaller than 0.7 μm × 0.7 μm square without a visible core were categorized as foci. Using ImageJ software, TDP-43-positive cells were manually inspected and quantified across three biological replicates, with a total of >250 TDP-43-positive cells counted. Quantification was reported as the percentage of total TDP-43-positive cells within each morphological category.

### Analysis of TDP-43 shell size in human and yeast cells

Live-cell imaging was performed as described under Fluorescence microscopy. To compare shell sizes between human and yeast cells, 30 shells were randomly selected from each group. Fluorescence intensity across individual condensates was measured in ImageJ along a defined line through the structure. The distance between the two intensity peaks was used to calculate shell diameter. Diameters from all shells were then compiled and plotted in Microsoft Excel to determine the average shell size in human and yeast cells.

### Analysis of chaperone-TDP-43 colocalization

Live-cell imaging was performed as described under Fluorescence microscopy. To assess chaperone enrichment within the core of TDP-43 shell-like condensates, fluorescence intensities of eGFP (TDP-43) and mCherry (chaperones) were measured along a defined line across the condensates, as detailed in each experiment. Fluorescence intensities were normalized to the maximum value within each measurement and plotted as relative fluorescence intensity (RFI) profiles. For colocalization analysis during early stages of TDP-43 expression (3 h post-induction), Z-stack images were acquired at 0.3 μm intervals to capture the possible dynamic movement of foci/condensates. Using ImageJ software, colocalization between TDP-43 and chaperones was manually inspected and quantified across three biological replicates, with a total of >300 TDP-43-positive cells counted. The same approach was used for late-stage TDP-43 expression (6 h post-induction). In both cases, cells were categorized into three groups: (1) cells containing multiphase condensates, (2) cells containing uniform foci colocalizing with chaperone signal, and (3) cells containing uniform foci lacking chaperone colocalization. Quantification was reported as the percentage of total TDP-43-positive cells within each category.

### Time-lapse imaging

Live-cell imaging was performed as described under Fluorescence microscopy. To investigate the dynamic properties of TDP-43 condensates, time-lapse imaging was used to capture condensate fusion events. Images of the same field were acquired every 1 s continuously for 5 min. The images were manually inspected to identify cells exhibiting real-time condensate fusion events. To track the maturation of TDP-43 from uniform foci to multiphase condensates, cells induced for 3 h, when the majority of the cells exhibited uniform foci, were used for microscopy. Z-stack images were acquired at 0.3 μm intervals every 2 min over a total duration of 30 min. Images were manually inspected to identify cells containing condensates undergoing maturation, and representative cells were selected for analysis. Fluorescence intensity measurements were performed along a defined line indicated in the corresponding figures to determine the formation of a hollowed core. Fluorescence intensities were normalized to the maximum value measured across all Z-stack images and plotted as relative fluorescence intensity (RFI) profiles. To examine chaperone recruitment during TDP-43 condensate maturation, cells induced for 3 h were imaged under similar conditions. Z-stack images were acquired at 0.2-μm intervals every 3 min for a total of 45 min. Images were manually inspected to identify cells with maturing condensates, and every Z-stack slice was analyzed to ensure that observed uniform foci/shell-like structures were not due to out-of-focus signal. Fluorescence intensities of eGFP (TDP-43) and mCherry (chaperones) were measured along defined lines, normalized to the maximum value across all Z-stack images, and plotted as RFI profiles accordingly.

### Time-point analysis of TDP-43 expression

To assess the state of TDP-43 condensation over time, cells were harvested at 2-, 3-, 4-, and 5-h post-induction and imaged as described under Fluorescence microscopy. Using ImageJ software, TDP-43-positive cells were manually inspected and quantified across three biological replicates, with a total of >500 TDP-43-positive cells counted. Cells were categorized as containing multiphase condensates or uniform foci. Quantification was reported as the percentage of total TDP-43-positive cells within each category. To examine the effect of halting TDP-43 expression, a similar approach was used. Glucose was added to stop TDP-43 expression, and cells were harvested at 0-, 1-, and 2-h after glucose addition. Quantification was performed using the same approach across three biological replicates, with a total of >350 TDP-43-positive cells counted.

### Fluorescence recovery after photobleaching (FRAP) analysis and quantification

FRAP experiments were performed on live-cell samples using a ZEISS LSM 980 microscope equipped with Airyscan two and a Plan-Apochromat 63× oil immersion objective with a numerical aperture of 1.4. Condensates ≥1 μm in diameter were selected for analysis. eGFP fluorescence was excited and photobleached using a 488 nm laser at 100% laser power with ∼10 iterations. Following photobleaching, time-lapse images were acquired at 1-s intervals using an attenuated laser power intensity of 0.5%.

Fluorescence intensities within the photobleached and unbleached regions were measured at each time point, *t*, using ImageJ. The relative fluorescence intensity at each time point (RFI(*t*)) was calculated as:

$$RFI\left(t\right)=\frac{I\left(t\right)\cdot I^{\prime }\left(Ref\right)}{I^{\prime }\left(t\right)\cdot I\left(Ref\right)}$$

Where *I*(*t*) and *I′*(*t*) represent the fluorescence intensity at time point *t* in the photobleached and unbleached region, respectively. *I*(*Ref*) and *I′*(*Ref*) represent the initial (pre-bleach) intensities of the photobleached and unbleached regions, respectively. The resulting RFI values were normalized between 0 and 1, where 0 corresponds to the minimum fluorescence intensity (immediately post-bleach), and one corresponds to the maximum observed intensity during recovery. These values were plotted to generate FRAP recovery curves. An average of 10 foci/condensates per experiment was used to calculate the mean and standard deviation.

## Data availability

This paper does not report any original code. Any additional information required to reanalyze the data reported in this paper is available from the corresponding author upon request (yjchoe@ntu.edu.sg).

## Supporting information

This article contains supporting information.

## Conflict of interest

The authors declare that they have no conflicts of interest with the contents of this article.
