# Supplementary material for: J-domain proteins cooperate with Hsp70 to drive multiphase separation of RNA-binding-deficient TDP-43
Source: J Biol Chem. 2025 Oct 22;301(12):110854. doi: 10.1016/j.jbc.2025.110854 (PMC12688062; doi:10.1016/j.jbc.2025.110854)
Supplement: Supporting Figures [file mmc1.pdf]

J-domain proteins cooperate with Hsp70 to drive multiphase separation of RNA-binding-deficient TDP-43

Kian Hua Yeo[1], Jian Hua Kong[1], Qing Hao Ng[1], Mi-Jeong Yoon[1], Olivia Agatha[1], Eunyoung Chae[2,3], Hyun O Lee[4], H Shawn Je[5], Young-Jun Choe[1]*

[1]School of Biological Sciences, Nanyang Technological University, Singapore 637551, Singapore.

[2]Department of Biological Sciences, National University of Singapore, Singapore 117558, Singapore.

[3]Department of Biology, University of Oxford, Oxford, United Kingdom.

[4]Department of Biochemistry, University of Toronto, Toronto, ON, M5S 1A8, Canada.

[5]Neuroscience and Behavioral Disorders Programme, Duke-National University of Singapore Medical School, 8 College Road, Singapore 169857, Singapore.

*Correspondence: yjchoe@ntu.edu.sg

Supporting information

Supplemental Figures and legends: Figure S1-S7

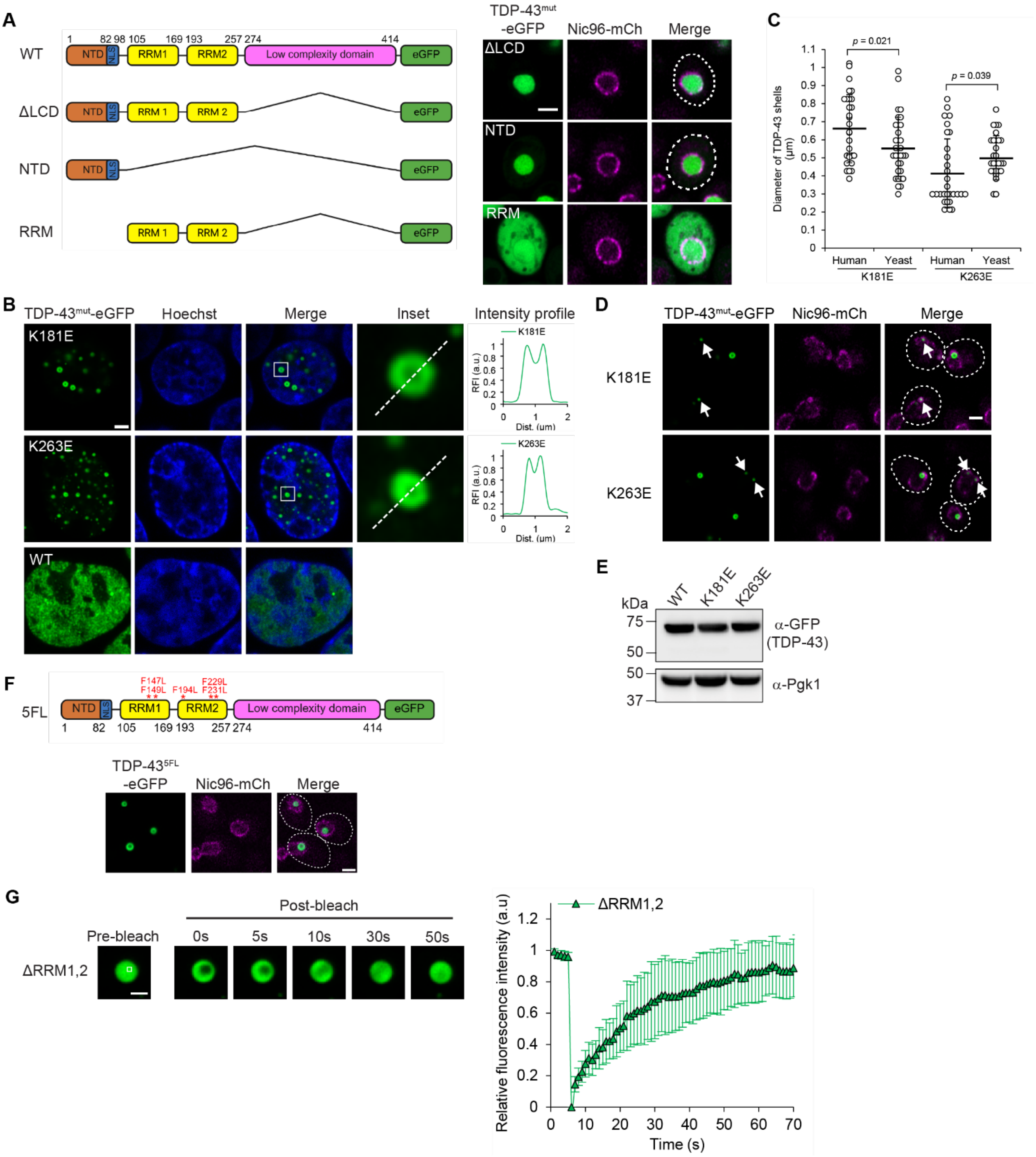
A
WT
ΔLCD
NTD
RRM
NTD
NLS
RRM1
RRM2
Low complexity domain
eGFP
TDP-43mut-eGFP
Nic96-mCh
Merge
C
Diameter of TDP-43 shells (μm)
p = 0.021
p = 0.039
Human
Yeast
K181E
K263E
B
Hoechst
Inset
Intensity profile
RFI (a.u)
Dist. (um)
D
E
kDa
α-GFP (TDP-43)
α-Pgk1
F
5FL
F147L
F149L
F194L
F229L
F231L
TDP-43[5FL]-eGFP
G
Pre-bleach
Post-bleach
ΔRRM1,2
Relative fluorescence intensity (a.u)
Time (s)

Figure S1. RNA-binding-deficient TDP-43 forms shell-like condensates. *A*, Schematic representation of truncated TDP-43 mutants (ΔLCD, NTD and RRM) tagged with eGFP at their C-termini, shown in the left panel. Microscopy images of each mutant TDP-43 expressed in yeast are shown in the right panel. Nic96-mCherry was co-expressed as a nuclear marker. Dashed lines indicate cell outlines. Scale bar, 2 μm. *B*, TDP-43^{K181E}-eGFP and TDP-43^{K263E}-eGFP were expressed under the CMV promoter in HEK293T cells. Areas within white squares are enlarged in the insets and analyzed for intensity profiles. Hoechst was used to counterstain the nuclei. Scale bar, 2 μm. *C*, Size comparison of shell-like condensates in human (HEK293T) and yeast cells. Each dataset represents measurements of 30 shell-like condensates. Data were collected from three human cells and 30 yeast cells, for both K181E and K263E. *p*-values were obtained by two-tailed Student's *t* test. *D*, TDP-43^{K181E}-eGFP and TDP-43^{K263E}-eGFP were expressed under the *GAL1* promoter for 6 hours in yeast. Both uniform foci and multiphase condensates were observed. Arrows indicate uniform foci. Dashed lines indicate cell outlines. Nic96-mCherry was used as a nuclear marker. Scale bar, 2 μm. *E*, Immunoblot analysis confirming the stability of TDP-43^{K181E}-eGFP and TDP-43^{K263E}-eGFP in yeast. Pgk1 was used as a loading control (n = 3). *F*, Schematic of the RNA-binding-deficient TDP-43 mutant (5FL) tagged with eGFP at the C-terminus (top). TDP-43^{5FL}-eGFP expressed in WT cells formed shell-like condensates (bottom). Shells were observed in 46 ± 5% of 5FL-expressing cells. Scale bar, 2 μm. *G*, FRAP analysis of ΔRRM1,2 condensates in yeast. Representative images of a condensate before and after photobleaching are shown in the left panel. The white square indicates the photobleached area. Scale bar, 1 μm. The fluorescence recovery (mean ± SD; n = 10 condensates) is shown in the right panel.

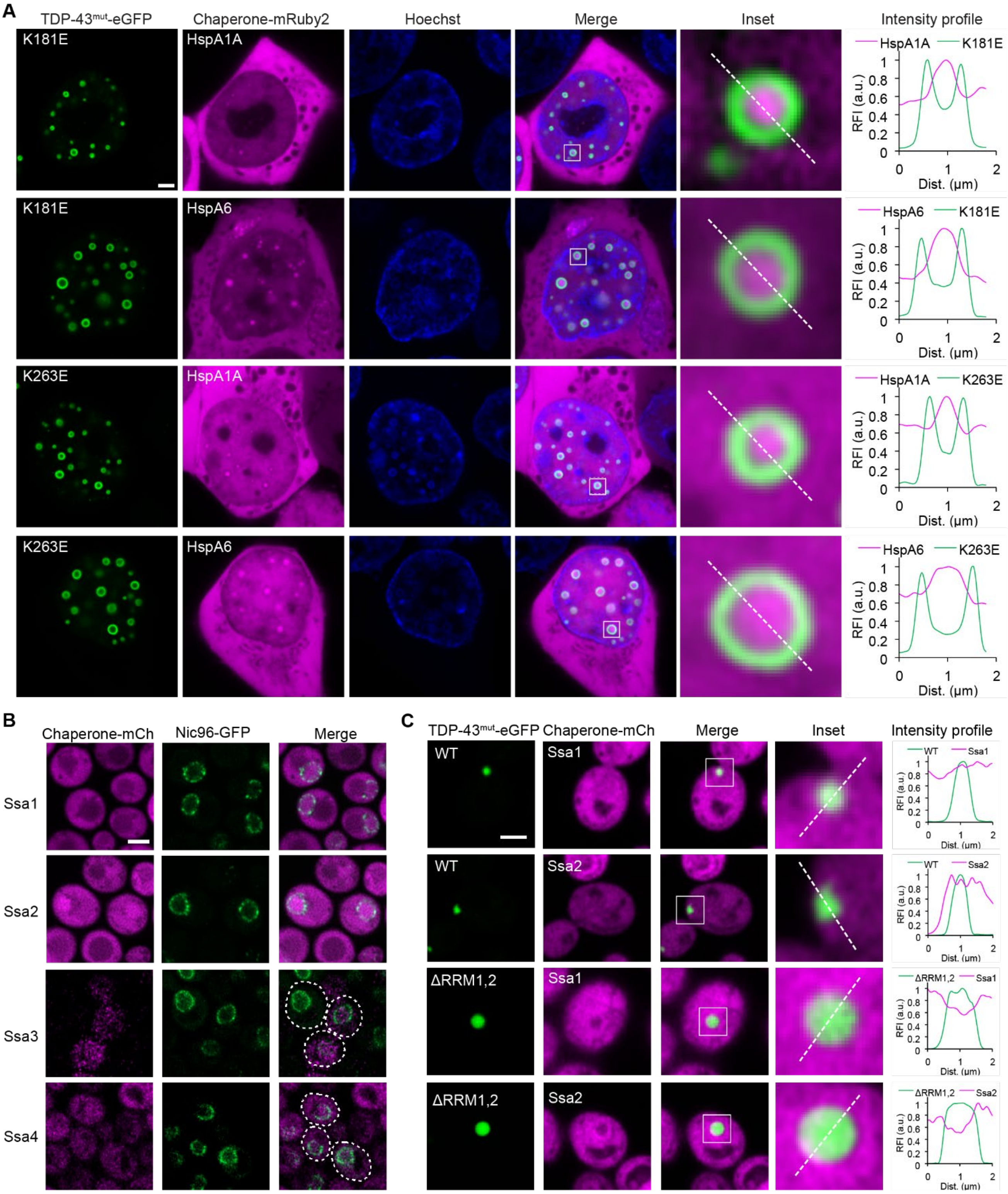
A
TDP-43mut-eGFP
Chaperone-mRuby2
Hoechst
Merge
Inset
Intensity profile
K181E
HspA1A
HspA6
K263E
RFI (a.u.)
Dist. (μm)
B
Chaperone-mCh
Nic96-GFP
Merge
Ssa1
Ssa2
Ssa3
Ssa4
C
TDP-43mut-eGFP
Chaperone-mCh
Merge
Inset
Intensity profile
WT
ΔRRM1,2
Ssa1
Ssa2

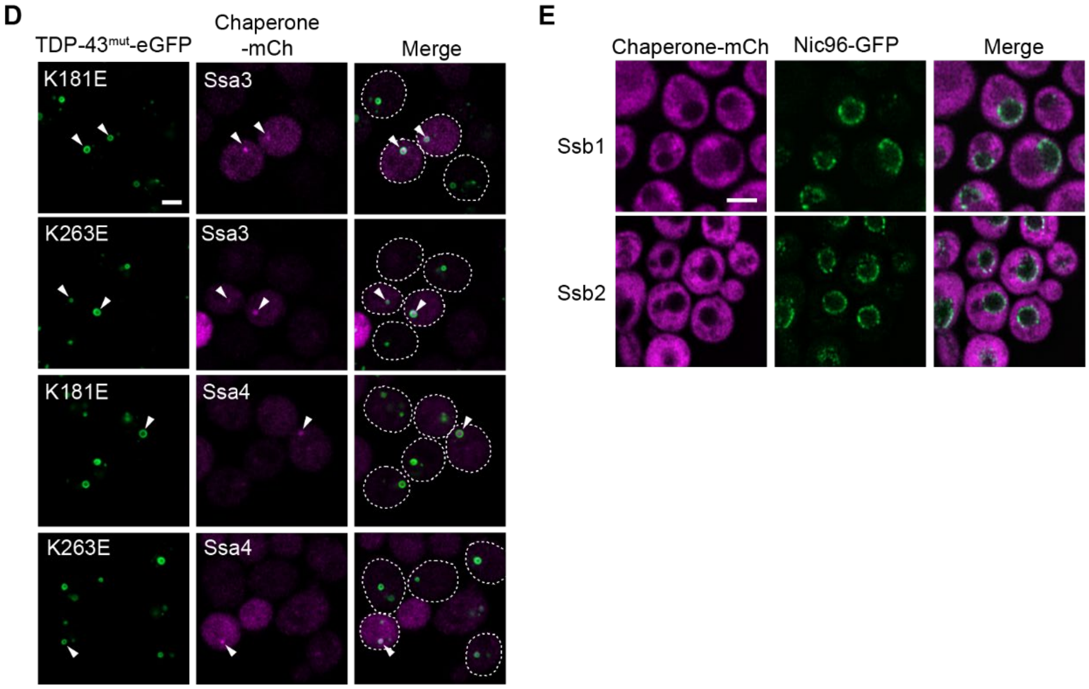

Figure S2. Multiphase separation of TDP-43 and Hsp70. *A*, Vectors expressing TDP-43^{K181E}-eGFP or TDP-43^{K263E}-eGFP were co-transfected with vectors expressing HspA1A-mRuby2 or HspA6-mRuby2 into HEK293T cells. HspA1A and HspA6 are human Hsp70 family members. Shells within the white squares are enlarged in the insets and analyzed for intensity profiles. Hoechst was used to counterstain the nuclei. Scale bar, 2 μm. *B***,** Representative microscopy images of Ssa subfamily members in yeast. Nic96-GFP was used as a nuclear marker. Scale bar, 2 μm. *C*, WT and ΔRRM1,2 TDP-43 proteins were expressed in yeast cells with genomic *SSA1* and *SSA2* tagged with C-terminal mCherry. Areas within white squares are enlarged in the insets and analyzed for intensity profiles. Scale bar, 2 μm. *D*, *SSA3* and *SSA4* were expressed at higher levels in some cells, showing enrichment in the cores of TDP-43 shells (arrowheads). Dashed lines indicate cell outlines. Scale bar, 2 μm. *E*, Representative microscopy images of Ssb subfamily members in yeast. Nic96-GFP was used as a nuclear marker. Scale bar, 2 μm.

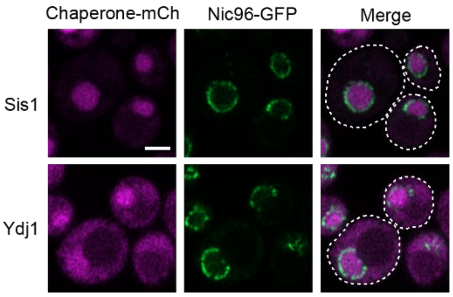

Figure S3. Localization of J-domain proteins (JDPs) in yeast cells. Nic96-GFP was expressed as a nuclear marker in cells with genomic *SIS1* or *YDJ1* tagged with C-terminal mCherry. Dashed lines indicate cell outlines. Scale bar, 2 μm.

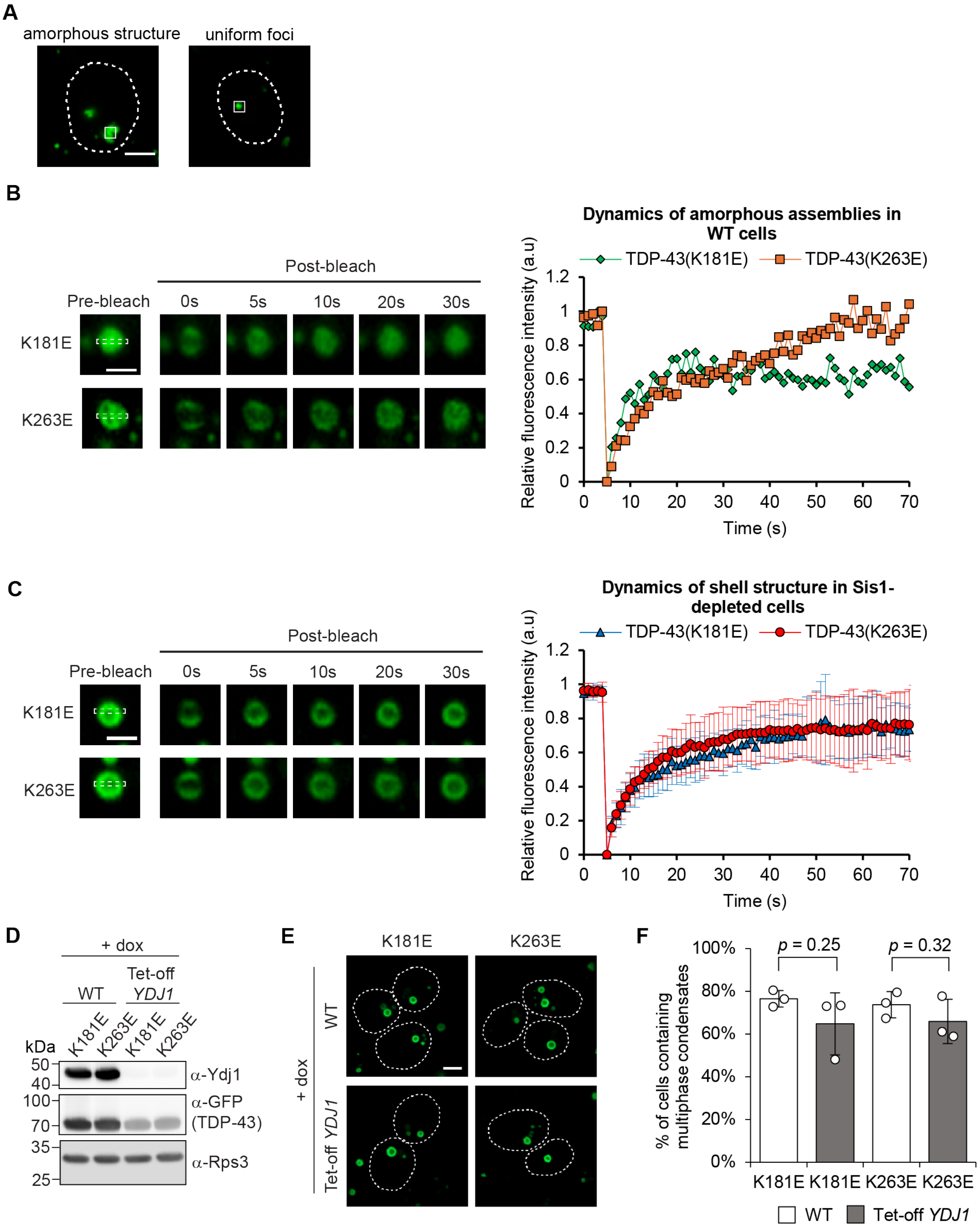

Figure S4. JDP Ydj1 is not required for TDP-43 multiphase separation. *A*, Amorphous structures were defined as irregularly shaped assemblies larger than a 0.7 μm × 0.7 μm square, while structures smaller than this reference were categorized as foci. A white square (0.7 μm × 0.7 μm) is shown for size comparison. Dashed lines indicate cell outlines. Scale bar, 2 μm. *B*, FRAP analysis of the rare amorphous TDP-43 assemblies observed in WT cells. Representative images of a condensate before and after photobleaching are shown (left panel). The dashed rectangle indicates the photobleached

area. Scale bar, 1 µm. *C*, FRAP analysis of TDP-43 shell structures observed in Tet-off *SIS1* cells. Cells were treated with doxycycline (10 µg/mL) for ~20 hours, followed by TDP-43 induction with galactose for 6 hours. The images of condensates before and after photobleaching are shown (left panel). The dashed rectangle indicates the photobleached area. The fluorescence recovery (mean ± SD; n = 10 condensates) is shown in the right panel. Scale bar, 1 µm. *D*, Immunoblot analysis confirming Ydj1 depletion upon doxycycline treatment. WT and Tet-off *YDJ1* cells are genetically identical, except that the endogenous *YDJ1* promoter in Tet-off *YDJ1* cells was replaced with a doxycycline-repressible promoter. Cells were treated with doxycycline (10 µg/mL) for ~20 hours, followed by TDP-43 induction with galactose for 6 hours. Rps3 was used as a loading control (n = 3). *E*, Representative images of TDP-43 shells, taken from the experiment shown in (*D*). Dashed lines indicate cell outlines. Scale bar, 2 µm. *F*, Quantitative analysis of RNA-binding-deficient TDP-43 shell formation in cells depleted of Ydj1. WT (white bars) and Tet-off *YDJ1* cells (gray bars), treated with doxycycline as described in (*D*), were compared. Data are presented as mean ± SD; n = 3 independent experiments; *p*-values were obtained by two-tailed Student's *t* test.

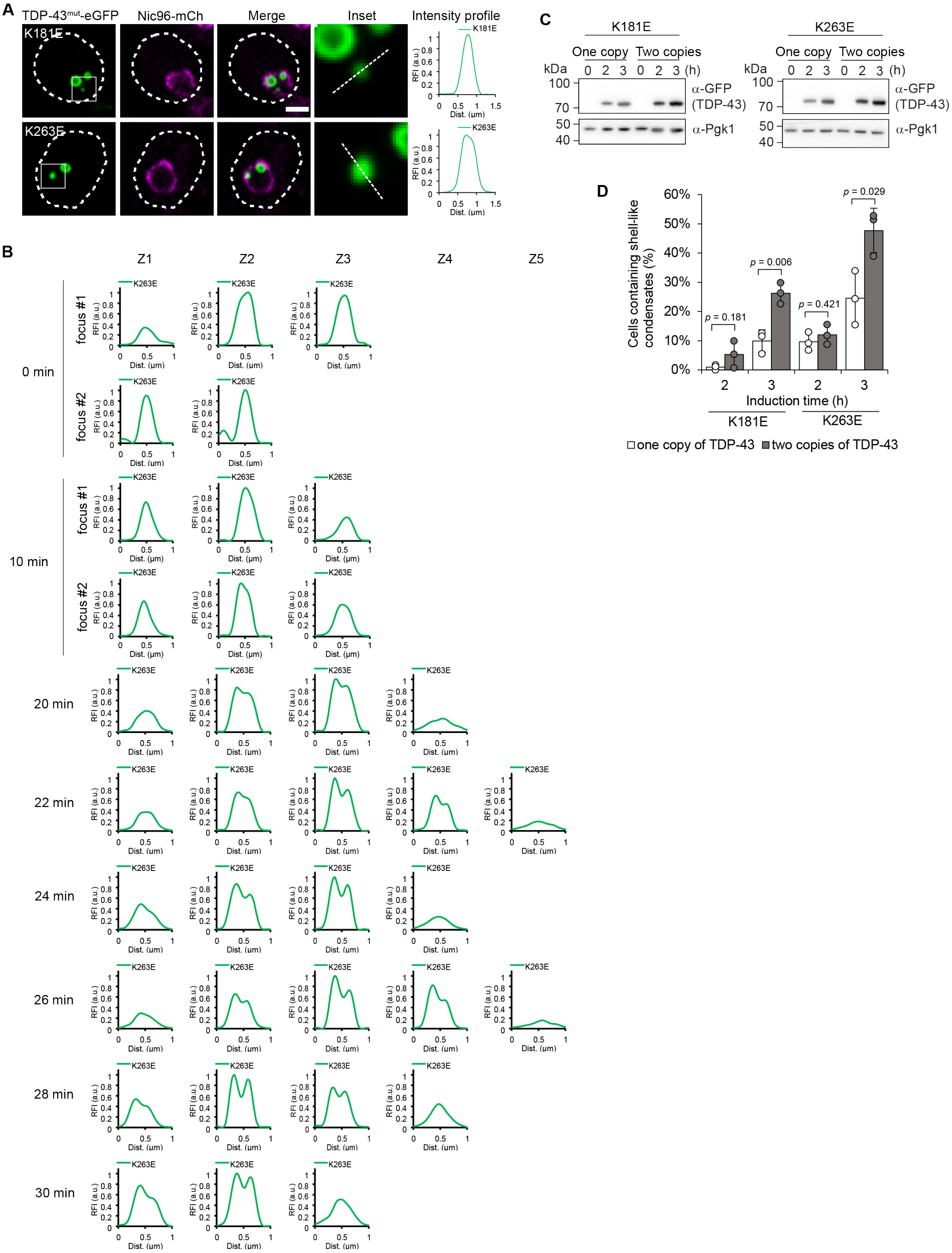

A
TDP-43mut-eGFP
Nic96-mCh
Merge
Inset
Intensity profile
K181E
K263E
RFI (a.u.)
Dist. (um)
B
Z1
Z2
Z3
Z4
Z5
0 min
10 min
20 min
22 min
24 min
26 min
28 min
30 min
focus #1
focus #2
Dist. (μm)
C
K181E
K263E
One copy
Two copies
kDa
0 2 3 0 2 3 (h)
α-GFP (TDP-43)
α-Pgk1
D
Cells containing shell-like condensates (%)
p = 0.181
p = 0.006
p = 0.421
p = 0.029
Induction time (h)
one copy of TDP-43
two copies of TDP-43

Figure S5. Transition of TDP-43 foci into multiphase condensates. *A*, RNA-binding-deficient TDP-43 mutants were expressed under the *GAL1* promoter for 6 hours. Uniform foci are enlarged in the insets and analyzed for intensity profiles. Dashed lines indicate cell outlines. Nic96-mCherry was used as a nuclear marker. Scale bar, 2 μm. *B*, Intensity profiles measured across the condensates shown in Fig. 5E at different Z-planes spanning the entire volume of the condensates, illustrating the transformation of a uniform focus into a multiphase condensate. *C*, TDP-43 expression levels were increased by introducing an additional copy of the expression plasmid (Two copies). For low-expression samples (One copy), a corresponding empty vector was co-transformed as a control. Expression levels of each TDP-43 mutant were examined by anti-GFP immunoblotting. Induction times are indicated in hours (h). Pgk1 was used as a loading control. *D*, Quantification of cells exhibiting shell-like condensates after 2 or 3 hours of galactose-induced expression of TDP-43 mutants. Data are presented as mean ± SD; >300 cells counted across n = 3 independent experiments.

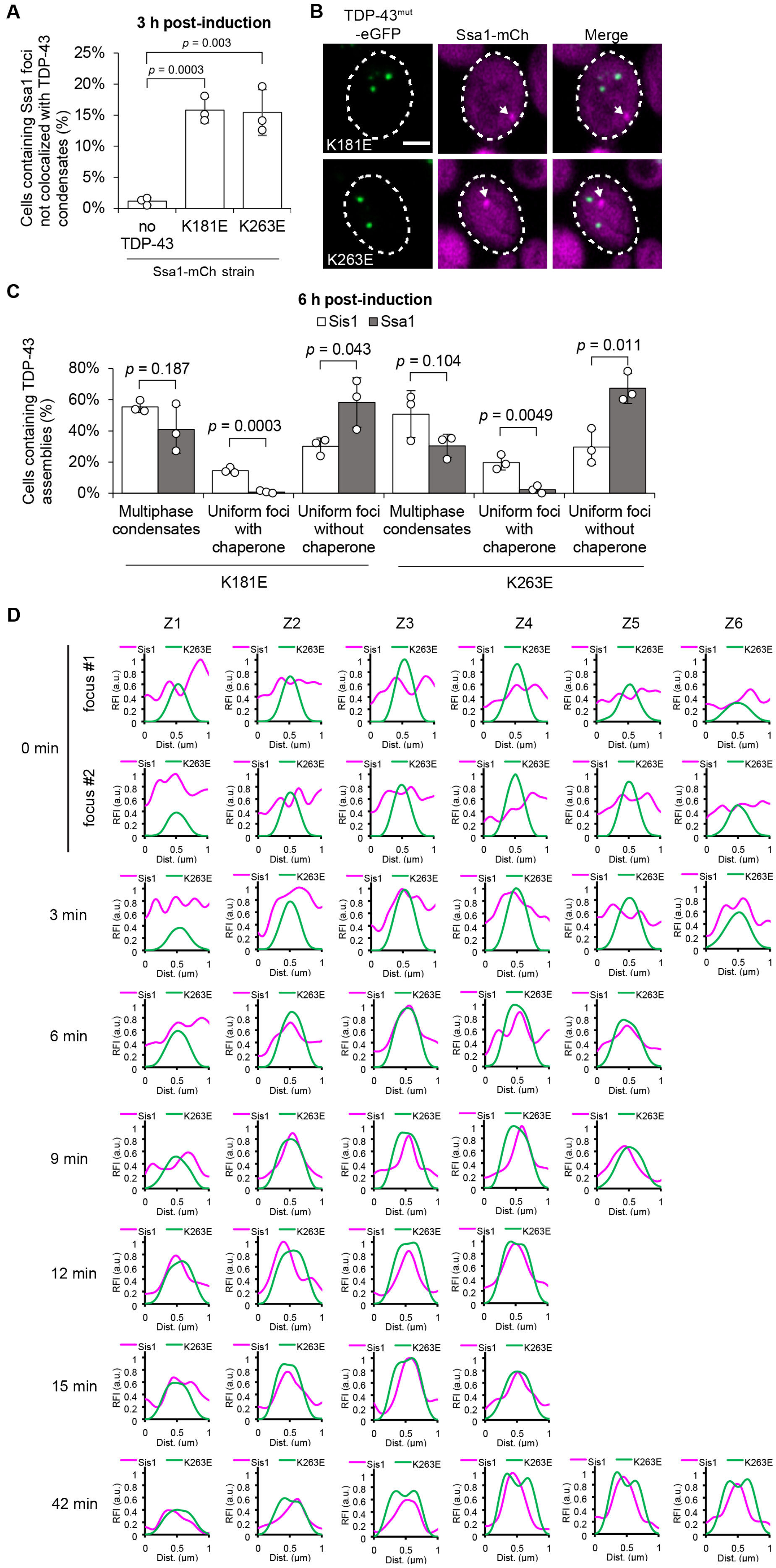
A
3 h post-induction
Cells containing Ssa1 foci not colocalized with TDP-43 condensates (%)
25%
20%
15%
10%
5%
0%
p = 0.003
p = 0.0003
no TDP-43
K181E
K263E
Ssa1-mCh strain
B
TDP-43mut -eGFP
Ssa1-mCh
Merge
K181E
K263E
C
6 h post-induction
Sis1
Ssa1
Cells containing TDP-43 assemblies (%)
80%
60%
40%
20%
0%
p = 0.187
p = 0.0003
p = 0.043
p = 0.104
p = 0.0049
p = 0.011
Multiphase condensates
Uniform foci with chaperone
Uniform foci without chaperone
K181E
K263E
D
Z1
Z2
Z3
Z4
Z5
Z6
0 min
focus #1
focus #2
3 min
6 min
9 min
12 min
15 min
42 min
Sis1
K263E
RFI (a.u.)
Dist. (μm)

E

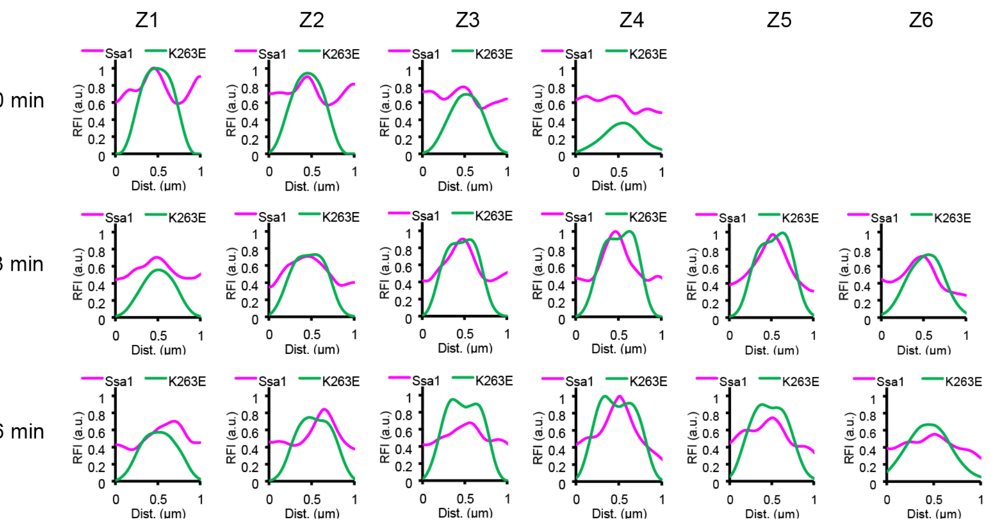

Figure S6. Sequential recruitment of chaperones during the transition of TDP-43 condensates. *A*, Quantitative analysis of microscopy data from Ssa1-mCherry cells expressing TDP-43^{mut}-eGFP at 3 hours post-induction, showing the formation of non-colocalized Ssa1 foci independent of TDP-43 foci. Data are presented as mean ± SD; n = 3 independent experiments; *p*-values were obtained by two-tailed Student's t test. *B*, Microscopy images of cells displaying Ssa1 foci that do not colocalize with TDP-43 foci at 3 hours post-induction. Arrows indicate Ssa1-mCherry puncta that do not colocalize with TDP-43. Dashed lines indicate cell outlines. Scale bar, 2 μm. *C*, Quantitative analysis of cells expressing TDP-43^{mut}–eGFP at 6 hours post-induction, examining the formation of chaperone-enriched TDP-43 foci. Data are presented as mean ± SD; n = 3 independent experiments; *p*-values were obtained by two-tailed Student's t test. *D–E*, Fluorescence intensity profiles across individual Z-planes of the condensates shown in Fig. 6E and Fig. 6F, spanning the entire volume of the condensates to examine the colocalization of chaperone with TDP-43.

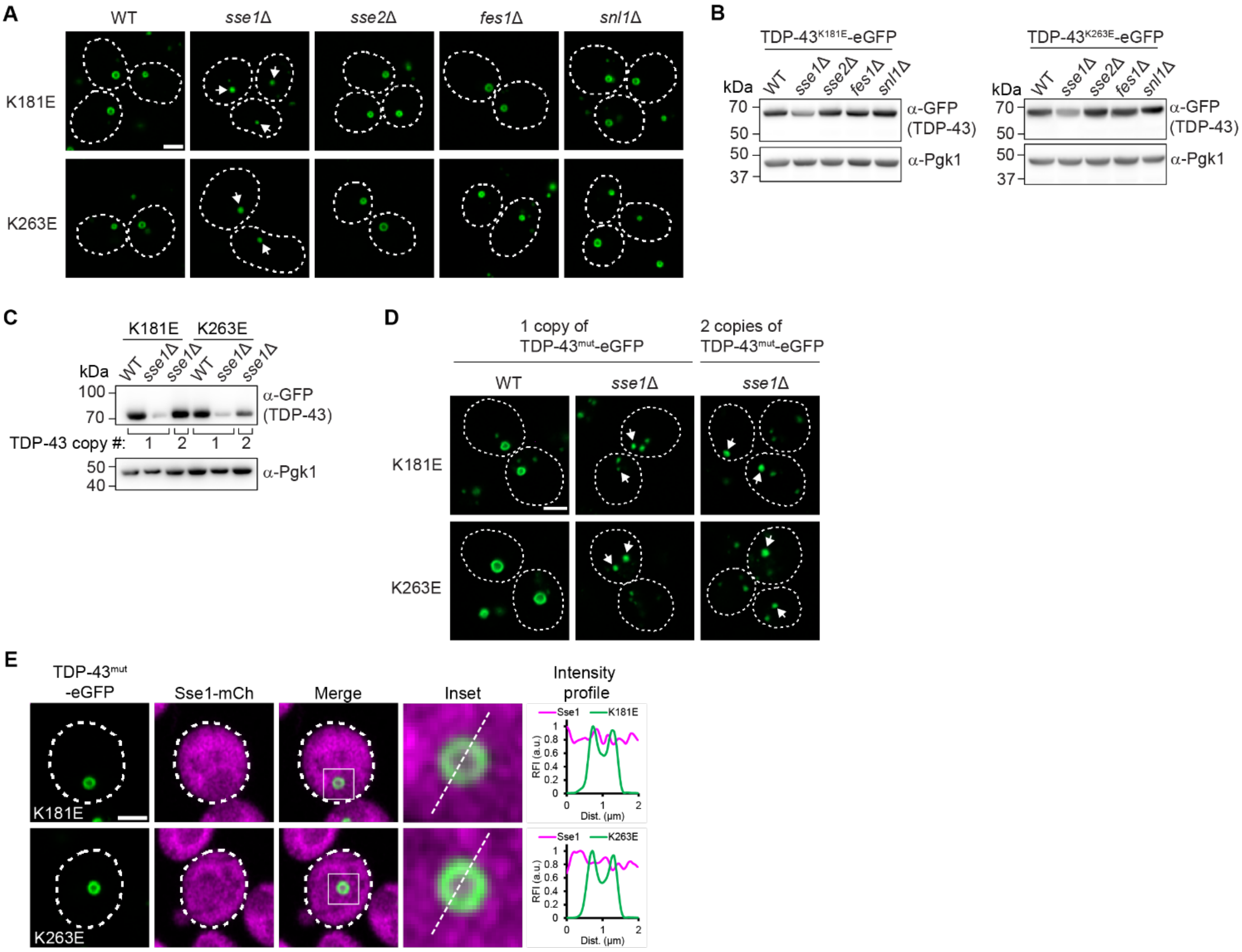

Figure S7. TDP-43 multiphase separation is severely impaired in *sse1*Δ yeast cells. *A*, K181E and K263E TDP-43^{mut}-eGFP were expressed under the *GAL1* promoter for 6 hours in each NEF deletion strain. Arrows indicate amorphous assemblies of TDP-43 in the *sse1*Δ mutant. Scale bar, 2 μm. *B*, TDP-43^{mut}-eGFP expression was induced as described in (*A*), and protein levels were monitored by anti-GFP immunoblotting. Pgk1 was used as a loading control. *C*, TDP-43^{mut}-eGFP expression levels were increased in *sse1*Δ cells by introducing an additional copy of the expression plasmid (copy # 2). For copy # 1 samples, a corresponding empty vector was co-transformed as a control. TDP-43-eGFP mutants were expressed under the *GAL1* promoter for 6 hours, and protein levels were examined by anti-GFP immunoblotting. Pgk1 was used as a loading control (n = 3). *D*, WT and *sse1*Δ cells expressing TDP-43^{mut}-eGFP from one or two copies of the expression plasmid (*GAL1* promoter, 6 h; as described in (*C*)) were examined by confocal microscopy. Scale bar, 2 μm. *E*, K181E and K263E mutants were expressed under the *GAL1* promoter for 6 hours in a yeast strain with genomic *SSE1* tagged at the C terminus with mCherry. Areas within white squares are enlarged in the insets and analyzed for intensity profiles. Scale bar, 2 μm.
